# Posttraumatic growth and posttraumatic stress – a network analysis among Syrian and Iraqi refugees

**DOI:** 10.1080/20008066.2022.2117902

**Published:** 2022-09-21

**Authors:** Samuli Kangaslampi, Kirsi Peltonen, Jonathan Hall

**Affiliations:** a Faculty of Social Sciences / Psychology, Tampere University, Tampere, Finland; b Department of Psychology, University of Turku, Turku, Finland; c INVEST Research Flagship Center, University of Turku, Turku, Finland; d Department of Peace and Conflict Research, Uppsala University, Uppsala, Sweden

**Keywords:** PTSD, posttraumatic growth, network analysis, refugee, war, trauma, TEPT, crecimiento postraumático, análisis de redes, refugiados, guerra, trauma, PTSD, 创伤后成长, 网络分析, 难民, 战争, 创伤

## Abstract

**Background:**

Traumatic events related to war and displacement may lead to development of posttraumatic stress symptoms (PTSS), but many war trauma survivors also report experiencing posttraumatic growth (PTG). However, the phenomenon of PTG remains poorly understood among refugees. Previous findings are also contradictory on whether more PTSS associate with PTG and what specific symptoms or aspects of growth may account for any possible link.

**Objective and Method:**

Here, we aimed to better understand posttraumatic growth among refugees, especially its structure and most important constituent elements, as well as how it associates with PTSS. We employed regression and network analysis methods with a large sample (*N *= 3,159) of Syrian and Iraqi refugees living in Turkey self-reporting on PTG and PTSS.

**Results:**

We found PTG and PTSS to be clearly distinct phenomena. Still, they often co-occurred, with a positive, slightly U-shaped relationship found between levels of PTSS and PTG. The main bridge between the constructs was identified from intrusive symptoms to having new priorities in life, although new priorities were more peripheral to the overall network structure of PTG. Meanwhile, discovering new psychological strengths and abilities and a new path in life emerged as elements most central to PTG itself.

**Conclusions:**

Many refugees report elements of PTG, even as they suffer from significant PTSS. The two phenomena appear distinct but positively associated, supporting the idea that intense cognitive processing involving distress may be necessary for growth after trauma. Our findings may inform efforts to support refugee trauma survivors in finding meaning and perhaps even growth after highly challenging experiences.

## HIGHLIGHTS

- Refugees from Syria and Iraq report substantial posttraumatic growth.
- Posttraumatic stress symptoms and posttraumatic growth positively associated.
- Intrusive symptoms appear as main link to posttraumatic growth.
- Discovering new strengths, abilities, and paths appears central to posttraumatic growth among refugees.

## Abstract (es)

**Antecedentes:** Los eventos traumáticos relacionados con la guerra y el desplazamiento pueden conducir al desarrollo de síntomas de estrés postraumático (SEPT), pero muchos sobrevivientes de traumas de guerra también informan que experimentan un crecimiento postraumático (CPT). Sin embargo, el fenómeno de CPT sigue siendo poco comprendido entre los refugiados. Los hallazgos previos también son contradictorios sobre si más SEPT se asocian con CPT y qué síntomas o aspectos específicos del crecimiento pueden explicar cualquier posible vínculo.

**Objetivo y Método:** Aquí, nuestro objetivo fue comprender mejor el crecimiento postraumático entre los refugiados, especialmente su estructura y los elementos constitutivos más importantes, así como también de que forma se asocia con los SEPT. Empleamos métodos de análisis de red y regresión con una muestra grande (*N* = 3159) de refugiados sirios e iraquíes que están viviendo en Turquía y que informan sobre CPT y SEPT.

**Resultados:** Encontramos que CPT y SEPT son fenómenos claramente distintos. Sin embargo, a menudo coincidieron, con una relación positiva, ligeramente en forma de U, encontrada entre los niveles de SEPT y CPT. El principal puente entre los constructos fue identificado desde los síntomas intrusivos a tener nuevas prioridades en la vida, aunque las nuevas prioridades eran más periféricas a la estructura de red general de CPT. Mientras tanto, el descubrimiento de nuevas fortalezas y capacidades psicológicas y un nuevo camino en la vida emergieron como elementos más centrales para el CPT en sí.

**Conclusiones:** Muchos refugiados reportan elementos de CPT, incluso mientras sufren de SEPT significativo. Los dos fenómenos parecen distintos pero asociados positivamente, apoyando la idea de que un procesamiento cognitivo intenso que involucre angustia puede ser necesario para el crecimiento después del trauma. Nuestros hallazgos pueden informar los esfuerzos para ayudar a los refugiados sobrevivientes de trauma a encontrar significado y tal vez incluso crecer después de experiencias altamente desafiantes.

## Abstract (zh)

**背景：**战争和流亡相关创伤事件可能导致创伤后应激症状 (PTSS) 的发展，但许多战争创伤幸存者也报告体验到了创伤后成长 (PTG)。然而，对难民中 PTG 现象仍然知之甚少。先前关于更多 PTSS 是否与 PTG 相关以及哪些特定症状或成长方面有任何可能连接上的研究结果有所矛盾。

**目的与方法：**在这里，我们旨在更好地了解难民的创伤后成长，尤其是其结构和最重要的构成元素，以及它如何与 PTSS 相关联。我们对一个居住在土耳其的叙利亚和伊拉克难民的大样本 (*N* = 3,159)中进行自我报告的 PTG 和 PTSS采用回归和网络分析方法。

**结果：**我们发现 PTG 和 PTSS 是明显不同的现象。尽管如此，它们经常同时发生，在 PTSS 和 PTG 水平之间发现呈正的、略呈 U 形的关系。构念之间的主要桥接被确定为从闯入症状到有新的生活优先事项，尽管新的优先事项在 PTG 整体网络结构中更为边缘。同时，发现新的心理力量和能力以及新的人生道路成为 PTG 本身最核心的元素。

**结论：**许多难民即使患有严重的 PTSS，还是报告了 PTG 元素。这两种现象看似不同但呈正相关，支持了涉及心理痛苦的强烈认知加工可能对于创伤后成长是必需的这一观点。我们的研究结果可能为支持难民创伤幸存者在极具挑战性的经历后寻找意义甚至成长的工作提供信息。

## 1. Introduction

evere traumatic events such as exposure to war may lead to substantial psychological disturbance for many trauma survivors, including the development of long-term posttraumatic stress symptoms (PTSS) (Blackmore et al., 2020; Morina et al., 2018). However, many people also report that facing and surviving a traumatic event has led to positive changes in some area of their lives, to the extent that their maturity, development, wisdom, or level of adaptation exceeds pre-trauma levels. Such positive changes have been termed posttraumatic growth (PTG; Tedeschi & Calhoun, 2004), and may include changed priorities and greater appreciation of life, closer relationships with others, improved sense of personal strength, recognition of new possible futures or life paths, and spiritual development (Tedeschi & Calhoun, 1996).

PTG may be a rather common experience, with one meta-analysis finding an average prevalence of 53% for moderate to high PTG after different traumatic events (Wu et al., 2019). It appears war-related traumatic events, too, may lead to the sort of positive changes termed posttraumatic growth. Substantial PTG has been reported among combat veterans (Greenberg et al., 2021; Whealin et al., 2020), Holocaust survivors (Greenblatt Kimron et al., 2019), refugees in general (Chan et al., 2016) and refugees from the Syrian civil war in particular (Acar et al., 2021; Ersahin, 2020). Meanwhile, PTSS reaching diagnostic levels have been reported for 15–31% of war-affected populations and refugees (Blackmore et al., 2020; Charlson et al., 2019; Morina et al., 2018).

### 1.1. Posttraumatic growth and posttraumatic stress symptoms

Crucially, experiencing PTG does not suggest that the traumatic event(s) were overall beneficial, nor that the trauma survivor has not suffered because of them. The same person may experience both distress, including PTSS, and growth, at the same or at different times after the traumatic event. In fact, PTG may be more likely when trauma survivors persist in thinking and talking about and trying to make sense of the trauma, that is, in intense cognitive processing (Henson et al., 2021; Tedeschi & Calhoun, 2004). Such processing often involves intrusive thoughts and the need to give up now unattainable goals and hopes. It may accordingly be experienced as difficult and distressing.

Indeed, across trauma types, meta-analyses have found more PTSS to correlate with more concurrent PTG (*r *= .22–.33) (Liu et al., 2017; Shakespeare-Finch & Lurie-Beck, 2014). However, there may be a limit to this positive association, as a curvilinear, inverse U-shape relationship between PTG and PTSS has been found in several recent studies among combat veterans (Greenberg et al., 2021; Na et al., 2021; Whealin et al., 2020) and in a meta-analysis of 42 studies with different types of trauma (Shakespeare-Finch & Lurie-Beck, 2014). This suggests PTG might be strongest when PTSS are moderate, rather than low or severe. In contrast, several studies have also found no positive association between PTSS and PTG (e.g. Peters et al., 2021; Powell et al., 2003; Thomas et al., 2021).

Trauma type could explain some of these discrepant findings. Two meta-analyses found the type of traumatic event experienced to moderate the relationship between PTSS and PTG (Liu et al., 2017; Shakespeare-Finch & Lurie-Beck, 2014), with highest correlations after conflict-related trauma. Further, a meta-analysis by Marziliano et al. (2020) found a robust but clearly smaller (*r* = .08) positive association between PTSS and PTG among cancer survivors and patients, as compared with survivors of conflict-related trauma.

Our study involves refugees from Syria and Iraq living in Turkey. A few previous studies have assessed posttraumatic growth in these specific refugee populations (Acar et al., 2021; Cengiz et al., 2018; Ersahin, 2020; Kılıç et al., 2016), although none have examined the dynamics between PTSS and PTG in more detail. Studying Syrian refugees in Turkey, Ersahin (2020) and Cengiz et al. (2018) found very high levels of PTSS and a modest positive relationship between self-reported PTSS and PTG. Acar et al. (2021) further found war exposure and life-threatening events to correlate with domains of PTG among Syrian refugees, while Kılıç et al. (2016), studying young Iraqi war survivors, found associations between trauma exposure and PTG to depend on the type of trauma, with adversity related trauma positively linked to PTG. In this study, we aim to further explore the dynamic interplay between symptoms and growth after war trauma by examining the precise links between particular types of PTSS and particular aspects or dimensions of PTG.

Our knowledge about links and associations at the domain or facet levels is limited and somewhat inconsistent. Based on four studies, Liu et al. (2017) found intrusion and hyperarousal, but not avoidance, symptoms to link to facets of PTG. Similarly, Greenberg et al. (2021) noted that re-experiencing symptoms were particularly connected to PTG among combat veterans. In contrast, studying older combat veterans with a longitudinal design, Whealin et al. (2020) found hyperarousal and avoidance symptoms to predict later PTG.

Regarding different aspects of PTG, Liu et al. (2017) found spiritual change, appreciation of life, new possibilities, and personal strength to associate with PTSS, but not relating to others. Meanwhile, Silverstein et al. (2018) noted the lowest correlations with PTSS for spiritual change and relating to others among undergraduate students exposed to different traumatic events. These findings are complicated by disagreement about the number of domains or factors into which PTG should be appropriately divided. Tedeschi and Calhoun (1996) suggested the five factors mentioned above. However, these domains may not be clearly distinguishable (Silverstein et al., 2018), and studies among refugees have found different three-factor solutions to fit better (Powell et al., 2003; Salo et al., 2005). Further, some researchers have suggested that in addition to a constructive and beneficial aspect, self-reported PTG might also include an illusory and maladaptive aspect, suggesting a two-component model of PTG (Schubert et al., 2016; Zoellner & Maercker, 2006). These contradicting findings and arguments call for more fine-grained examination of the structure of PTG and the specific links between areas of PTG and particular symptoms.

### 1.2. Network analysis of posttraumatic growth

We use network analysis (Borsboom & Cramer, 2013; Schmittmann et al., 2013) to help us answer some of the open questions about the nature of PTG and about how PTG associates with different PTSS. While various studies have assessed the network structure of PTSD in different populations (reviewed by Birkeland et al., 2020), network analyses of PTG are fewer. In the first one published, Bellet et al. (2018) found discovering a new path for life and greater personal strength to be elements most central to the network structure of PTG among bereaved college students. To our knowledge, just two studies have included both PTG and PTSS in a network analysis, both among Chinese survivors of a natural disaster. For Chinese adults who had lost a child in an earthquake, Peters et al. (2021) found finding a new path in life, greater sense of closeness with others, and ability to do better things with life to be most central to the network structure of PTG. In their analyses, changed priorities had almost no connection to the other elements of PTG, and was more associated with PTSS. Yuan et al. (2021) studied a larger sample of Chinese college students exposed to a typhoon and found changed priorities and stronger religious faith on the PTG side and intrusive thoughts and physiological reactivity on the PTSS side to be the most important bridges between the two constructs. These two studies were carried out in a context and with a population that is quite different from the refugees we are studying, however. To our knowledge, no previous network analyses of PTG, with or without considering PTSS, exist among refugees.

### 1.3. Present study

Here, we examine the associations between PTSS and PTG, the network structure of aspects of PTG, and the most important bridges between PTSS and PTG at the symptom and item level, among refugees displaced by the wars in Syria and Iraq and currently residing in Turkey. First, we use correlational and regression analyses to examine the linear and quadratic association between PTSS and PTG. Then, we estimate a network of elements of posttraumatic growth, which allows us to examine how it’s different aspects link to each other and which aspects appear as most central to the phenomenon. Finally, we add in types of PTSS into the network and study the links, whether positive or negative, between particular elements of PTG and PTSS. Insights gleaned may aid clinicians in supporting, or at least not hindering, PTG, and help us understand the phenomenon of PTG among refugees.

## 2. Method

### 2.1. Participants

The sample consists of 3159 refugees from Syria and Iraq residing in eleven cities in Turkey, mainly in the Central Anatolia region. Table 1 presents demographic details for the sample. Participants were mostly young adults (36.5% between 18 and 24 years of age), and relatively highly educated (36.6% more than 12 years of school). Around half of the participants were from Syria (49.9%) and half from Iraq (46.2%). Somewhat more participants were male (53.1%) than female (44.9%).

**Table 1.** Demographic details of the sample of Syrian and Iraqi refugees.

	*n*	%
*Gender*		
Male	1678	53.1
Female	1417	44.9
NA	64	2.0
*Age group*		
18–24	1153	36.5
25–34	877	27.8
35–44	480	15.2
45–54	308	9.7
55–74	219	6.9
75 or older	22	0.7
NA	100	3.2
*Home country*		
Syria	1575	49.9
Iraq	1460	46.2
NA	124	3.9
*Education*		
No formal education	106	3.4
<6 years of education	141	4.5
6 years of schooling	319	10.1
9 years of schooling	618	19.6
12 years of schooling	739	23.4
>12 years of schooling	1156	36.6
NA	80	2.5
*Potentially traumatic experiences*		
Lack of food or water	1424	45.1
Ill health without medical care	1086	34.4
Lack of shelter	1090	34.5
Imprisonment	621	19.7
Physical abuse	686	21.7
Serious injury	460	14.6
Combat situation	910	28.8
Indiscriminate shelling or bombing	2148	68.0
Being close to death	1042	33.0
Forced evacuation	1341	42.5
Forced separation from family	889	28.1
Murder of family or friend	979	31.0
Unnatural death of family or friend	735	23.3
Murder of stranger	715	22.6
Kidnapped	366	11.6
Tortured	435	13.8

Note: *N* = 3159.

### 2.2. Procedure and measures

A team of local assistants, themselves refugees from Syria and Iraq, were recruited and trained to administer the questionnaires. The use of local assistants in the administration of the study helped ensure that the study was carried out with cultural sensitivity and in a context of interpersonal trust. Building on trust networks established through fieldwork, we generally opted for community-based sampling, whereby existing participants recruited future participants from among their social networks, a sampling procedure often used to identify otherwise hidden populations. To supplement this recruitment strategy and diversify the sample, research assistants also approached refugees in breadlines, outside aid organisations, in public transportation hubs, at universities, and near refugee camps during daytime.

The same procedure for participant recruitment was carried out in eleven cities – Adana, Ankara, Antalya, Antakya, Balıkesir, Eskişehir, Istanbul, Kahramanmaraş, Konya, Mersin, and Yalova. To reach a large and diverse sample, the data were collected in three waves, in 2016, 2017, and 2019. Further details about data collection are provided in the Supplementary Material.

**Posttraumatic stress symptoms** were assessed using the Arabic translation of the six-item short form of the PTSD Checklist – Civilian version (PCL-C; Lang et al., 2012; Lang & Stein, 2005). This version of the checklist is based on DSM-IV diagnostic criteria for PTSD. See Supplementary Material for details on localisation of materials and measures. Participants evaluated ‘how much you have been bothered by each problem in the last month’ on a scale from 1 (*not at all*) to 5 (*extremely*). Sample items include ‘Avoid activities or situations because they remind you of a stressful experience from the past’ and ‘Feeling distant or cut off from other people’. We used the total sum of the six items, with a range of 6–30, as an index of overall symptom severity. A sum score of 14 or greater may be considered screening positive for PTSD (Lang & Stein, 2005). Estimates indicated good to excellent internal consistency (*α*_ordinal_ = .90, *ω_h_* = .82, *ω*_total_ = .94).

**Posttraumatic growth** was assessed using the Arabic translation of the 10-item short form of the Posttraumatic Growth Inventory (PTGI-SF; Cann et al., 2010). Participants evaluated the ‘degree to which this change occurred in your life as a result of all that has happened’ on a scale from 0 (*not at all*) to 5 (*to a very great degree*). Sample items include ‘I am able to do better things with my life’ and ‘I know better that I can handle difficulties.’ We used the total sum of the ten items, with a range of 0–50, as an index of overall posttraumatic growth. Estimates indicated good to excellent internal consistency (*α*_ordinal_ = .89, *ω_h_* = .78, *ω*_total_ = .91).

### 2.3. Missing data

There were a total of 4057/50544 (8.0%) answers missing on the PTGI-SF or the PCL-C. For descriptive statistics and examining correlations between PTSS and PTG, we used personal mean value imputation for up to three missing values in the PTGI-SF and up to one missing value in the PCL-C. For network analyses, all available data were used with no imputation.

### 2.4. Statistical analyses

We first explored the relationships between PTG and PTSS with correlational analyses and by examining plots with best fitting linear and quadratic relationships based on regression analyses. We then used network analysis to get more insight into the relationships between specific aspects of PTG and between aspects of PTG and classes of PTSS.

We followed the standards for estimating network structures and their accuracy and stability proposed by Epskamp et al. (2018) and the reporting standards suggested for cross-sectional data by Burger et al. (2020). We estimated Gaussian graphical models where edges and their associated coefficients represent a partial correlation between two nodes conditioned on all other included variables. We estimated both networks with PTG items only and combined PTG-PTSS networks. As the data consisted of Likert-type items with few response options, we used Spearman correlation matrices as input. We used the ggmModSelect function of the *qgraph 1.6.9* R package (Epskamp et al., 2012) to estimate unregularized Gaussian graphical models, using stepwise model selection to maximise the extended Bayesian information criterion. Simulation studies indicate model search rather than regularisation methods may be preferable for large samples (Epskamp, 2018; Williams & Rast, 2020). However, for easier comparison with earlier research and as sensitivity analyses, we also estimated the networks using the EBICglasso function, with hypertuning parameter γ set to 0.5. These alternative networks are presented in the Supplementary Material.

For making inferences about the networks, we used node strength and expected influence as metrics for node centrality. In the combined PTG-PTSS network, we further used the bridge strength and bridge expected influence metrics (Jones et al., 2019) as measures of bridge centrality – the degree to which particular nodes act as bridges between PTG and PTSS.

We assessed the stability and accuracy of the estimated networks using the *bootnet 1.4.3* package (Epskamp et al., 2018). We calculated 95% confidence intervals around the edge weights and expected influences using bootstrapping with 3000 bootstrapped samples. We then ran the edge-weights difference test and the expected influence difference test for each network. We further estimated correlation-stability coefficients for edge weights and estimates of expected influence using the case-dropping subset bootstrap method (Epskamp et al., 2018).

We carried out all analyses using R 4.0.5 (R Core Team, 2021) and a variety of R packages (listed in the Supplementary Material). We present the complete R scripts used for carrying out the analyses, as well as additional statistics in the Supplementary Material. The research data are available upon request from the third author.

### 2.5. Ethical issues

Uppsala University Ethical Review Board approved this project. Informed consent to collect data and use it for research was obtained from all participants. They were informed that participation was completely voluntary and that they could discontinue their involvement at any time for any reason. Participants received a small sum of money (10–20 Turkish lira) as compensation.

## 3. Results

### 3.1. Descriptive statistics

Table 2 presents mean levels and standard deviations for different aspects of PTG and different PTSS. Stronger religious faith and discovering that one is stronger than one thought before were the most reported aspects of PTG, while having a better understanding of spiritual matters and learning how wonderful people were least reported. Intrusions and reactivity to trauma reminders were the most reported PTSS, while difficulties concentrating were the least reported type of symptom. Levels of PTSS were high on average (*M* = 17.17), with 2001/3159 (63.3%) of participants scoring at or above the suggested cutoff for probable PTSD, based on DSM-IV criteria. Participants also reported moderate to high levels of posttraumatic growth on average (*M* = 32.32).

**Table 2.** Means and standard deviations for different aspects of posttraumatic growth and different types of posttraumatic stress symptoms for refugees from Syria and Iraq.

	*M*	SD	Abbreviation
*Aspects of posttraumatic growth*			
1 – Changed Priorities about What Is Important	3.06	1.37	ChangedPriorities
2 – Greater Appreciation for Value of Life	3.29	1.24	AppreciateLife
3 – Able to Do Better Things	3.32	1.22	AbleDoBetterThings
4 – Better Understanding of Spiritual Matters	2.98	1.42	BetterSpiritual
5 – Greater Sense of Closeness with Others	3.23	1.19	Closeness
6 – Established New Path for Life	3.19	1.29	NewPath
7 – Know Better I Can Handle Difficulties	3.32	1.16	BetterHandle
8 – Stronger Religious Faith	3.55	1.31	StrongerFaith
9 – Discovered I'm Stronger than I Thought	3.35	1.24	DiscStronger
10 – Learned How Wonderful People Are	2.98	1.32	WonderfulPeople
Total	32.32	8.65	
*Posttraumatic stress symptoms*			
1 – Intrusions	3.20	1.12	Intrusions
2 – Reactivity	3.06	1.16	Reactivity
3 – Avoidance	2.76	1.28	Avoidance
4 – Distance from Other People	2.73	1.35	Distance
5 – Irritability	2.76	1.22	Irritability
6 – Difficulties Concentrating	2.65	1.32	DiffConc
Total	17.17	5.93	

Note: *N *= 3159. Posttraumatic growth measured with the PTGI-SF on a scale of 0–5 and posttraumatic stress symptoms on the 6-item PCL-C on a scale of 1–5.

### 3.2. Correlational and regression analyses

**Figure 1.**
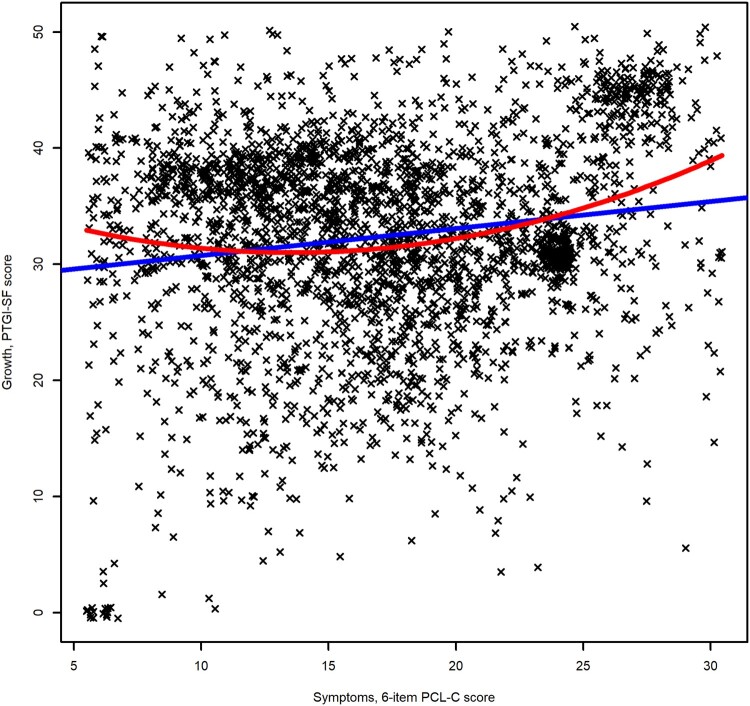
Relationship between posttraumatic stress symptoms and posttraumatic growth among Syrian and Iraqi refugees residing in Turkey. Best fitting linear and quadratic relationship presented in blue and red, respectively. Position of individual data points jittered for easier interpretation.

The overall relationship between PTG and PTSS is illustrated in Figure 1. Total symptom severity correlated positively with posttraumatic growth (*r *= .16, 95% CI [.13, .20], *p* < .0001). A slightly curvilinear (U-shaped) relationship between PTSS and PTG was a better fit than a strictly linear one. A regression model with both linear and quadratic PTSS terms predicting PTG explained a total of 4.2% of the variance in PTG (R^2 ^= .042, F(2,2858) = 63.12, *p* < .0001), see Supplementary Material for details.

Different categories of PTSS were differently associated with PTG. Intrusion (*r* = .23, 95% CI [.20, .27], *p* < .0001) and reactivity symptoms (*r *= .19 [.16, .23], *p* < .0001) correlated most strongly with total PTG, while irritability (*r *= .04 [−.0007, .07], *p *= .055) was the only symptom not exhibiting significant correlation. Of the aspects of posttraumatic growth, having new priorities in life (*r *= .26, 95% CI [.22, .29], *p *< .0001) and appreciating life more (*r *= .15, [.11, .18], *p *< .0001) had the highest correlations with total PTSS, while discovering one is stronger than one thought had the lowest correlation (*r *= .04, [.00, .08], *p* < .05). See Supplementary Table 1 for details.

### 3.3. Network structure – posttraumatic growth

**Figure 2.**
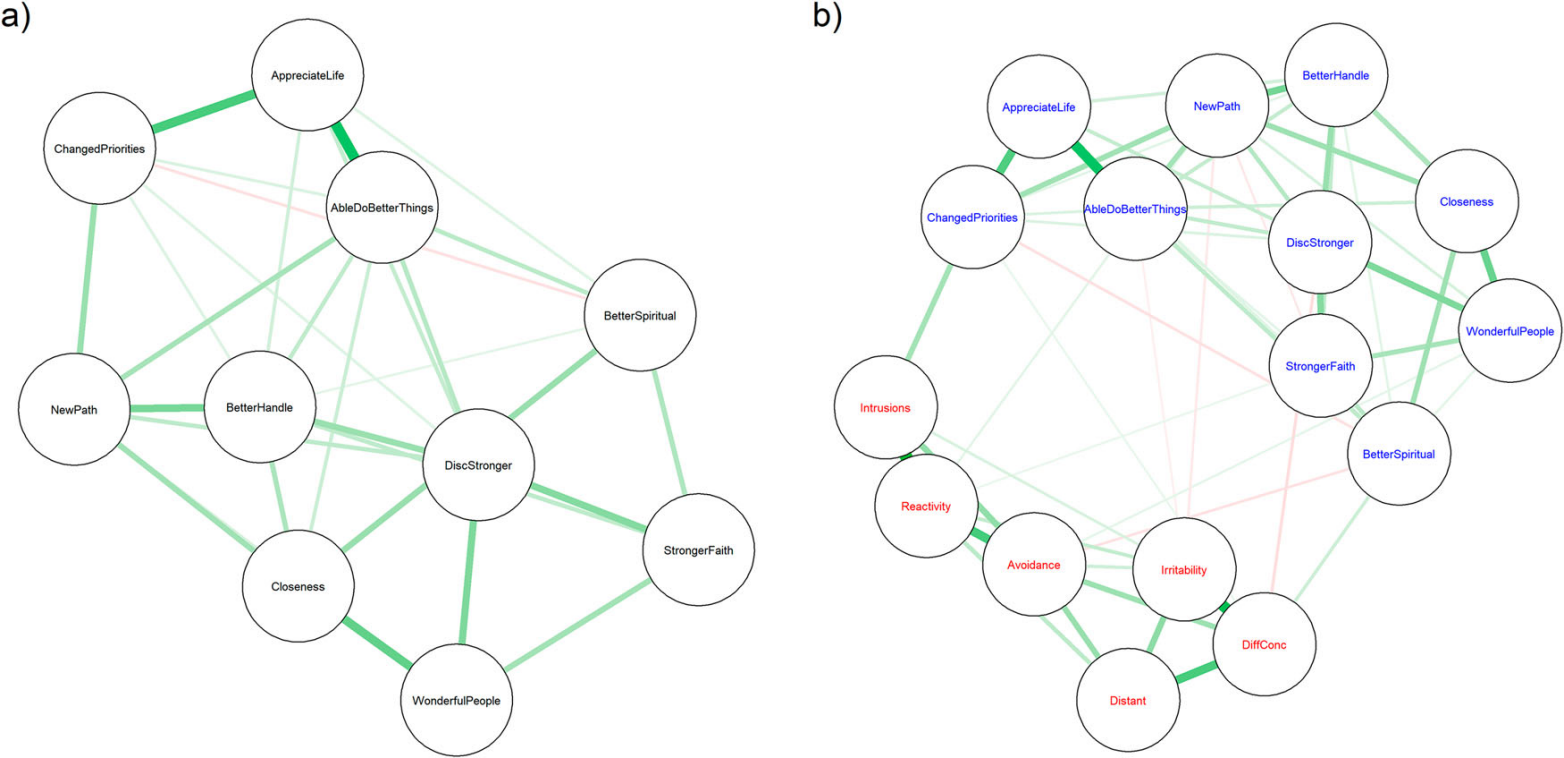
Gaussian graphical models of (a) aspects of posttraumatic growth and (b) aspects of posttraumatic growth (presented in blue) and types of posttraumatic stress symptoms (presented in red), for Syrian and Iraqi refugees residing in Turkey. Edge thickness represents degree of association as partial correlations, green edges indicate positive association, and red edges indicate negative association. Model selection estimation used for estimating the network and the *spring* algorithm used for layout determination.

The estimated network structure for aspects of PTG only is presented in Figure 2(a). The model selection algorithm returned a relatively dense network, with 29/45 non-zero edges. Still, mean edge weight was quite low (.09), and even the highest edge weight (between appreciating life and being able to do better things) was only .35. The other strongest edges were between appreciating life more and changed priorities (.30), and between increased closeness and realising how wonderful people are (.27). A single non-zero negative edge was estimated, between changed priorities and better understanding of spiritual things (−.07).

### 3.4. Network structure – posttraumatic growth and posttraumatic stress symptoms

The estimated network structure including both aspects of PTG and PTSS is presented in Figure 2(b). PTG and PTS elements emerged as clearly separate clusters, although there were a few links between the two. The network was somewhat less dense than the PTG only network, with 54/120 non-zero edges. Mean edge weight was again low (.06), although the highest edge weight was somewhat higher (.45, between intrusion and reactivity symptoms) than in the PTG only network. The second strongest edge was also identified between different PTSS, irritability and difficulties concentrating (.39). The highest edge weight for a link between PTSS and PTG was between changed priorities and intrusive symptoms (.17). Several weak negative edges were also identified, the strongest between discovering one is stronger than one thought and difficulties concentrating (−.08).

### 3.5. Inference – posttraumatic growth

**Figure 3.**
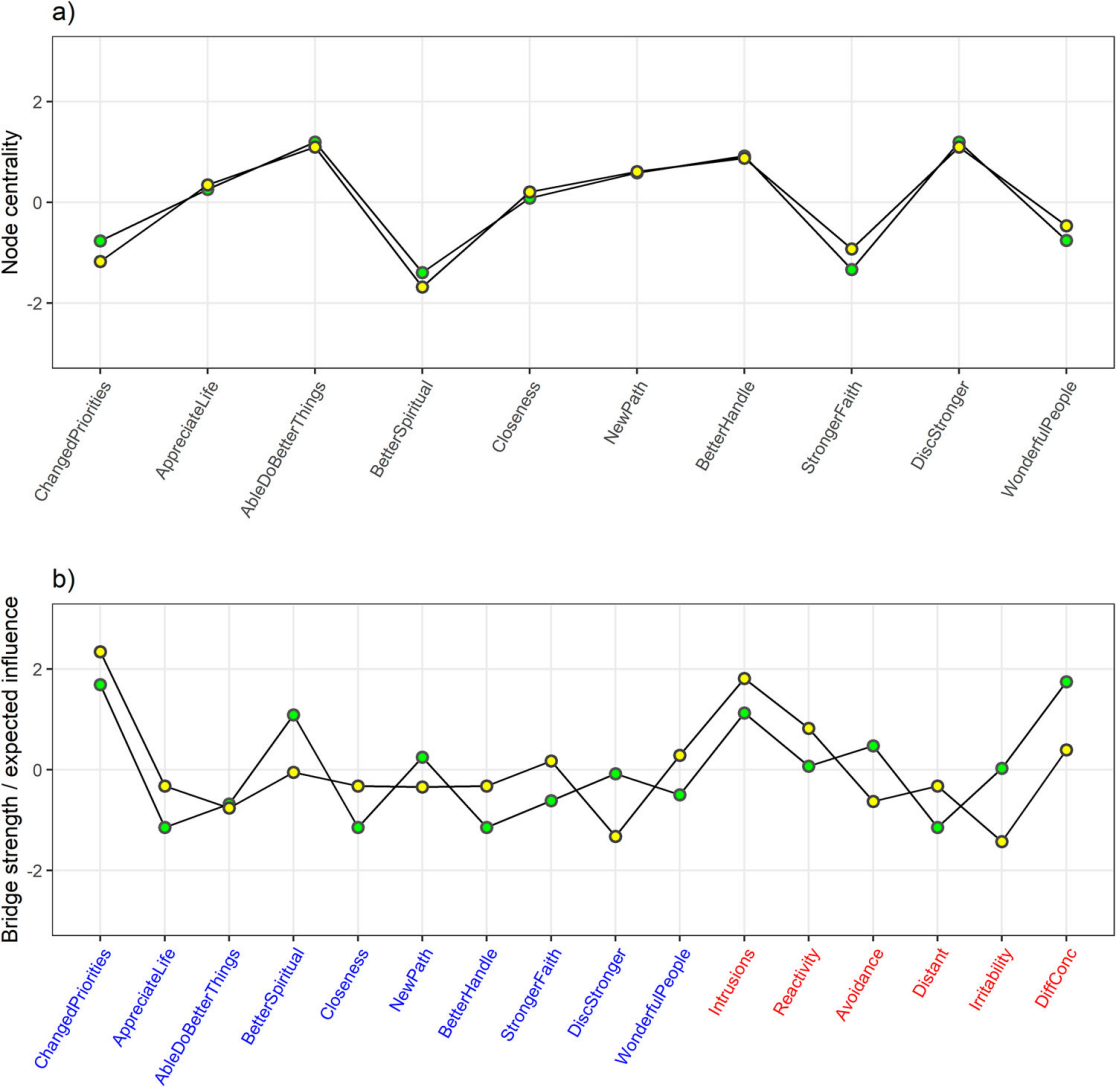
Node/bridge strength (green) and node/bridge 1-step expected influence (yellow) as measures of node/bridge centrality for (a) network of aspects of posttraumatic growth and (b) network of aspects of posttraumatic growth (presented in blue) and types of posttraumatic stress symptoms (presented in red), for Syrian and Iraqi refugees residing in Turkey. Scaled values presented.

Figure 3(a) presents scaled node strength and expected influence as centrality estimates for the PTG only network. Scaled and raw centrality indices are further presented in the Supplementary Material. Discovering one is stronger than one thought and being able to do better things emerged as the most central nodes (scaled/raw node strength 1.20/1.06, scaled/raw first-step expected influence 1.10/1.06, for both), while being able to better handle difficult situations was also central (0.92/1.01, 0.88/1.01). The religious items, having better understanding of spiritual matters (−1.39/0.64, −1.68/0.51) and stronger religious faith (−1.33/0.65, −0.93/0.65), were the least central to the PTG only network.

### 3.6. Inference – posttraumatic growth and posttraumatic stress symptoms

Of particular interest in the combined network were nodes that might act as bridges between PTS and PTG items. Scaled bridge strength and bridge expected influence are presented in Figure 3(b). They were highest for the PTG aspect of having new priorities in life (scaled/raw bridge strength 1.69/0.21, scaled/raw 1-step bridge expected influence 2.34/0.21) and for intrusive symptoms (1.13/0.17, 1.81/0.17). Bridge strength was also relatively high for better understanding of spiritual matters (1.09/0.16) and difficulties concentrating (1.75/0.21), but bridge expected influence was much lower for these nodes (−0.05/0.02 and 0.39/0.06, respectively), as they also had negative edges connecting them to the other side. Scaled node strength and expected influence as centrality estimates for the combined PTG-PTSS network are presented graphically, and scaled and raw node and bridge centrality indices are further provided in the Supplementary Material.

### 3.7. Accuracy and stability

Results of accuracy and stability analyses are presented in Supplemental Figures 1–4. Networks appeared to be accurately estimated, as confidence intervals around edge weights were moderately sized, all edges with weight estimates above 0.1 were statistically significantly different from 0, and many edge weight estimates significantly differed from each other. However, for all the identified negative edges, the bootstrapped confidence interval did include zero. Differences in edge weights were clearer for the combined PTG-PTSS network than for the PTG only network. The bootstrapped confidence intervals around expected influence were also moderately sized and most estimates differed significantly from each other.

Correlation-stability coefficients in the PTG only network were .88 for edge weights and > .90 for expected influence, while in the combined PTG-PTS network, they were > .90 for edge weights, and .88 for expected influence, all above the suggested threshold of 0.50 (Epskamp et al., 2018).

## 4. Discussion

We examined posttraumatic growth among Syrian and Iraqi refugees and links between aspects of posttraumatic growth and posttraumatic stress symptoms. It appears self-reported PTG is not an uncommon phenomenon among refugees with conflict-related traumatic experiences and active posttraumatic stress symptoms. Refugees reported an overall moderate to high level of posttraumatic growth, with stronger religious faith, discovering that one is stronger than one thought before, being able to do better things with one’s life, and knowing better that one can handle difficulties being the most endorsed aspects of PTG. Meanwhile, the participants also reported high levels of posttraumatic stress symptoms on average, especially intrusions and reactivity to trauma reminders.

We observed an overall positive, modest correlation between PTSS and PTG. However, a slightly U-shaped relationship was a better fit to the data than a strictly linear one. This stands in contrast to several recent studies among combat veterans (Greenberg et al., 2021; Na et al., 2021; Whealin et al., 2020) and the majority of earlier studies (Shakespeare-Finch & Lurie-Beck, 2014) that reported an inverted U-shape relation and stronger correlations overall. Considering the overall low to moderate strength of the observed associations, clearly other (psychological, social, cultural) factors may be more important for determining the level of PTG experienced and may explain these differences, but our findings do at least speak against any hard limit of PTSS too severe for the possibility of PTG.

### 4.1. Network structure of posttraumatic growth

We were able to estimate stable and apparently accurate networks of aspects of PTG. The estimated PTG only network was relatively dense. There was no evidence of strong clustering or particular aspects of PTG being set apart from the others. Some of the strongest links identified do correspond to the factor structure originally suggested for the PTGI-SF measure (Cann et al., 2010), especially the factors of relating to others and appreciating life. However, links between items thought to belong to the factors of spiritual change, personal strength, and new possibilities did also stand out.

Reviewing qualitative studies, Şimşir Gökalp and Haktanir (2021) argued for the universality of the phenomenon of PTG among refugees from many cultural backgrounds and identified four central themes in how refugees perceived such growth: improved psychological functioning, enhanced interpersonal relationships, reconstruction of the meaning of life, and positive future direction. These themes correspond somewhat but not fully to the domains of PTG suggested by Tedeschi and Calhoun (2004). Our findings on the structure of PTG among Syrian and Iraqi refugees especially highlighted the centrality of aspects of growth that fit the themes of improved psychological functioning – discovering one is stronger than one thought and knowing that one is better able to handle difficulties – and of positive future direction – being able to do better things with one’s life and establishing a new path in life. We could tentatively suggest that these are areas of special importance in determining whether refugees are able to find meaning in their hardship and experience posttraumatic growth.

Religious items, having better understanding of spiritual matters and having stronger religious faith, were the least central to the PTG network. Stronger religious faith is particularly interesting, as it was the most frequently endorsed PTG item, but had one of the lowest estimates for centrality to the network structure. This suggests that though many of the refugees had experienced increasing faith, such increases were not necessarily associated with other aspects of PTG.

Learning about how wonderful people are was also markedly low in centrality in our analyses and was among the most rarely endorsed items. As refugees, the participants in our study had escaped their home country because of the terrible actions of other people, so it is not surprising that any psychological growth they might experience as a result of their hardships would not rest crucially on learning about the benevolence of others. Refugees may further experience discrimination in the host country, which might further undermine this aspect of PTG. However, positive experiences in the host country or in relation to one’s loved ones could still be positive sources of growth and meaning making for some.

The role of changed priorities as part of PTG is also worth considering. In contrast to Peters et al. (2021) who studied earthquake survivors, we did not find changed priorities to be totally set apart from other aspects of PTG. Still, its centrality to was relatively low, which appears to be a stable feature of the network structure of PTG, as changed priorities was also among the least central items in the analyses by Bellet et al. (2018) and Yuan et al. (2021). As discussed below, this could reflect the diverse ways this item could be interpreted.

Overall, the network structure we identified for PTG among refugees appears more similar to that presented by Bellet et al. (2018) among bereaved university students than the one presented by Peters et al. (2021) for middle-aged parents who had lost a child. Age could be a factor, as most of our participants were young adults below 35 years of age. Even compared to Bellet et al. (2018), the PTG network we estimated had lower centrality for the items relating to spirituality, religion, and other people being wonderful. This could reflect aspects of posttraumatic adjustment unique to refugees or to the social and cultural context of the Middle East. Other recent research also calls for particular attention to potential cultural differences in the spiritual growth aspect of PTG (Garrido-Hernansaiz et al., 2022).

### 4.2. Combined network structure of posttraumatic growth and posttraumatic stress symptoms

We were able to estimate a stable and apparently accurate network for aspects of PTG and different types of PTSS together. The resulting network was less dense, and PTG and PTSS emerged as separate clusters. This suggests posttraumatic growth and posttraumatic stress are separate, clearly distinguishable phenomena among refugees, and not just two sides of the same coin. Still, a few, modest links between them were also identified. A main interest of ours was identifying which symptoms or aspects of PTG might act as crucial bridges over to the other cluster. Bridge centrality estimates were highest for having new priorities in life and intrusive symptoms.

The finding that intrusive symptoms were the main positive bridge to PTG is in accordance with the idea that PTG may require intense cognitive processing of the trauma and even rumination about the event and its meaning (Henson et al., 2021; Tedeschi & Calhoun, 2004). Previous studies with different approaches have also found intrusive symptoms to have the strongest link to PTG (Greenberg et al., 2021; Liu et al., 2017). Considering its low centrality in the PTG network, it is interesting that the changed priorities item was the main bridge between PTG and PTSS, and had the highest correlation to overall PTSS. If we were to exclude the changed priorities item, as Peters et al. (2021) did due to it not associating with other PTG items, the PTG and PTSS clusters would be far less interconnected, and intrusive symptoms would not connect over to PTG (see Supplementary Material for details).

The question about new priorities in what is important in life is certainly open to many interpretations by trauma survivors. As Peters et al. (2021) note, it could have quite a different, more negative connotation for those whose path in life has been drastically, forcefully altered. For some refugees, the new, changed priorities could relate to ensuring survival and well-being in a new environment or the need to practically re-construct one’s life in a new place. Understandably, such priorities may not be as linked to aspects of PTG as the psychological reconstruction of assumptions and worldview thought to be central to enabling PTG (Henson et al., 2021). If a significant number of participating refugees were thinking of new priorities related to, e.g. survival or vigilance here, this could explain some of the specific link between intrusive symptoms and changed priorities. On-going intrusions and re-experiencing the trauma might especially elevate and keep up the priority and importance given to remembering what happened, staying vigilant, ensuring basic survival, or getting help. A more positive interpretation, further supported by the link between changed priorities and appreciating the value of life more, is that processing the trauma intensely or being reminded of it often may promote realising what really matters in one’s life.

Interestingly, better understanding of spiritual matters and difficulties concentrating also had relatively high bridge strength, representing the total links they have with the other cluster, regardless of sign, but some of these links were negative. As the identified negative links were weak and uncertain, we are reluctant to interpret them, but our findings do point to a complex relationship between aspects of spiritual change and distress after traumatic events and may reflect both negative and positive forms of religious coping (Ano & Vasconcelles, 2005). We should note that religious belief and being active in religious practice was very common in our sample overall. This could affect our results, compared with environments where base levels of spirituality and religious observance are lower, such as in the studies by Yuan et al. (2021) and Peters et al. (2021).

### 4.3. Strengths and limitations

The large sample with a balanced representation of the genders is a major strength of this study. Community-based sampling and established trust networks allowed us to conduct this study among a difficult to reach population. Still, the sample was not random nor perfectly representative of refugees in the region. Reliance on mainly community-based sampling might mean that refugees with fewer social connections and perhaps poorer functioning had less chance of being recruited. This could bias the results in the direction of more reported PTG.

As for other limitations, our data were wholly cross-sectional, so we cannot make credible claims about the direction of possible causal effects and have only examined associations. The study was not pre-registered, so it is exploratory by nature. The use of a very brief measure for PTSS resulted in a coarse level of analysis for PTSS themselves. However, we do consider this level of analysis reasonable for examining links between PTSS and aspects of PTG.

Some adjacency effects appeared to be present, so that strongest edges / highest correlations occurred between subsequent items on surveys. Such order effects may be particularly problematic for network analyses (Trachik et al., 2020). As Trachik et al. (2020) did for PTSS, comparing results obtained by random and fixed item orders would be valuable for network analyses of PTG as well.

As we noted above, there is some disagreement about whether PTG as defined and measured using instruments like the PTGI-SF represents (only) truly adaptive and constructive changes after a traumatic event, or whether some aspects of it are more appropriately seen as illusions and wishful thoughts trauma survivors employ to cope with negative consequences or avoid confronting them (Schubert et al., 2016; Zoellner & Maercker, 2006). Interpreting and especially applying our findings to practice should take this possibility into account. Detailed analyses of PTG at the facet level as we have attempted here may contribute somewhat to clarifying the issue and separating these two potential sides of the phenomenon. However, future research on the topic would benefit from measures that explicitly account for this possibility, from qualitative analyses, and from linking different aspects of self-reported PTG to other mental health and well-being outcomes beyond PTSS.

## 5. Conclusions

This study reinforces the view that posttraumatic growth does not mean the absence of negative consequences of trauma (Shakespeare-Finch & Lurie-Beck, 2014). Among refugees, too, experiences of posttraumatic growth appear fairly common, and can co-exist with posttraumatic stress symptoms. Higher levels of symptoms, and of intrusive symptoms in particular, appear to associate with higher reported posttraumatic growth. Such findings underscore the role of cognitively processing and trying to come to terms with traumatic events, even though distressing, in experiencing psychological growth after hardships. Meanwhile, they also remind us about the importance of seeking and supporting elements of growth when working with traumatised refugees, even when they also report on-going symptoms.

Posttraumatic growth is not a unitary phenomenon, but includes several facets of psychological growth, maturity, and change. This study tentatively suggests that elements related to improved psychological functioning and to feelings of a positive future direction may be most crucial to experiences of psychological growth after adversity among refugees. On the other hand, spiritual growth and increased faith, while frequently experienced, may be a somewhat distinct phenomenon among refugees already quite religious on average. Regardless of type of trauma, the finding that changed priorities in what is important in life may be less central to PTG has now been repeated in several studies.

## Supplementary Material

Supplemental Material

Supplemental Material

Supplemental Material

Supplemental Material

Supplemental Material

Supplemental Material

Supplemental Material

Supplemental Material

Supplemental Material

## Data Availability

Data used in this research are available from the third author upon request. As permission to share data publicly was not requested from participants and the data includes potentially sensitive information, the data cannot be made fully publicly available. All scripts used to carry out data manipulation and statistical analyses are provided in the Supplementary Material.
