# Supplementary material for: Posttraumatic growth and posttraumatic stress – a network analysis among Syrian and Iraqi refugees
Source: Eur J Psychotraumatol. 2022 Sep 21;13(2):2117902. doi: 10.1080/20008066.2022.2117902 (PMC9518504; doi:10.1080/20008066.2022.2117902)
Supplement: Supplemental Material [file ZEPT_A_2117902_SM9051.docx]

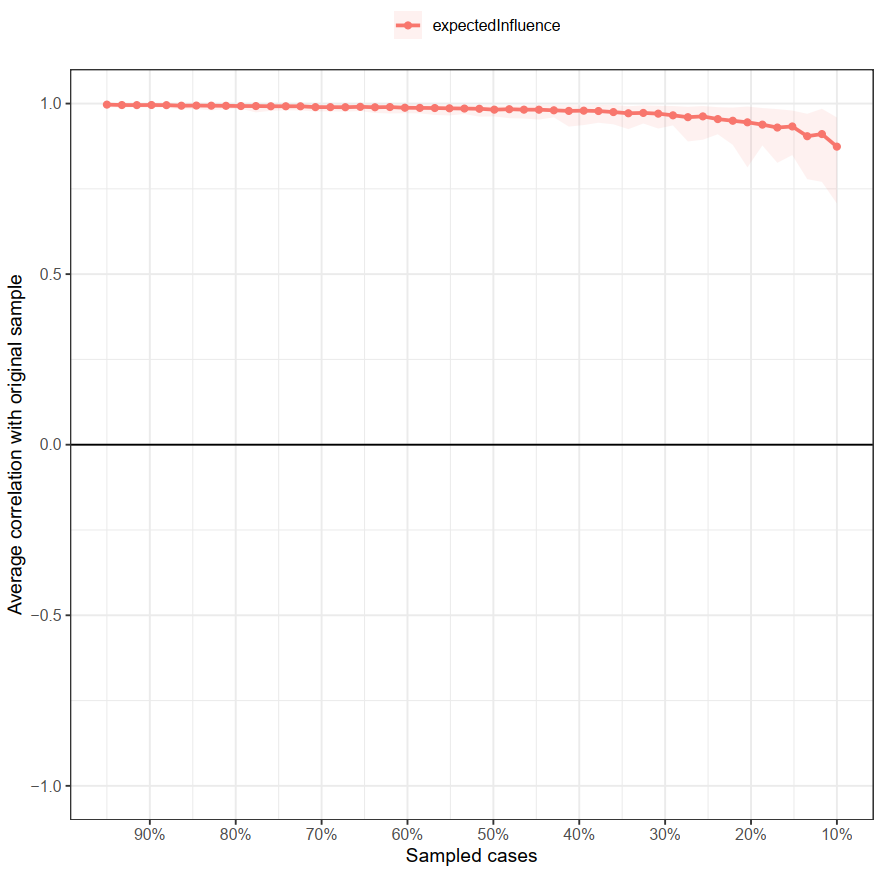


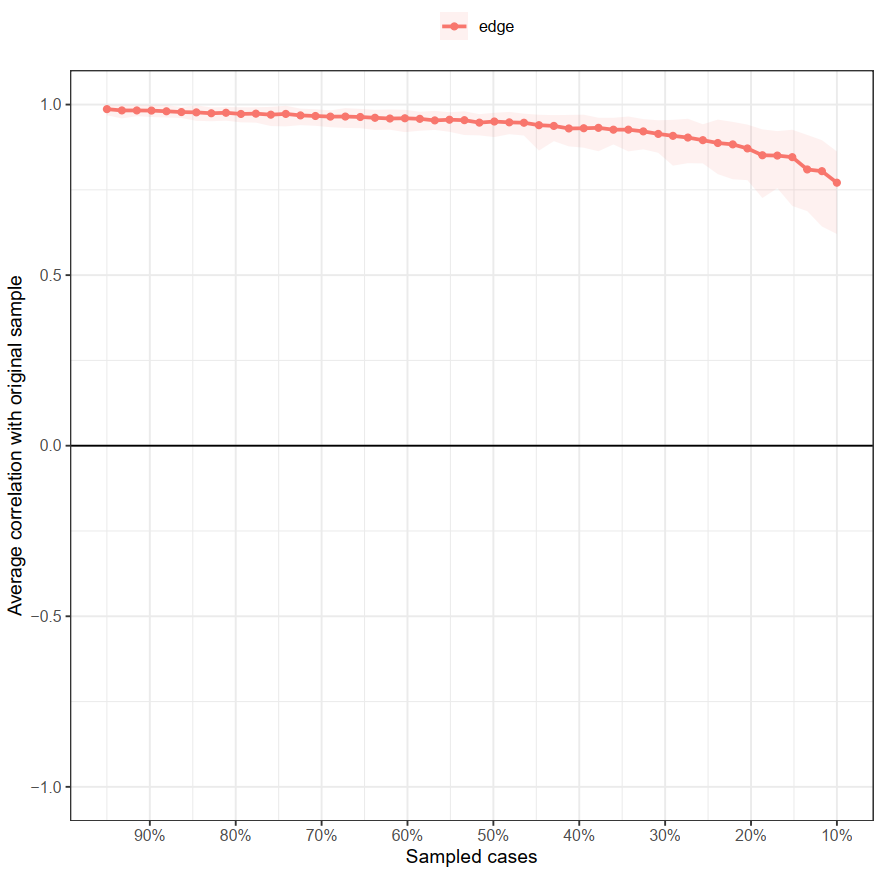
**a) b)**

**Supplementary Fig 2a-b.** Stability of estimated edge weights and expected influence metrics for network analysis of elements of posttraumatic growth among Syrian and Iraqi refugees residing in Turkey. Graphs of average correlation with original sample vs. share of sampled cases for increasing levels of case-dropping presented, based on 3000 bootstrapped samples of 50 sampling levels and a maximum of 90% of cases dropped for a) edge weights and b) expected influence. Correlation-stability coefficient (share of cases that could be dropped while maintaining correlation above .7 in at least 95% of samples) .88 for edges and > .90 for expected influence.
