# Supplementary material for: Posttraumatic growth and posttraumatic stress – a network analysis among Syrian and Iraqi refugees
Source: Eur J Psychotraumatol. 2022 Sep 21;13(2):2117902. doi: 10.1080/20008066.2022.2117902 (PMC9518504; doi:10.1080/20008066.2022.2117902)
Supplement: Supplemental Material [file ZEPT_A_2117902_SM9048.docx]

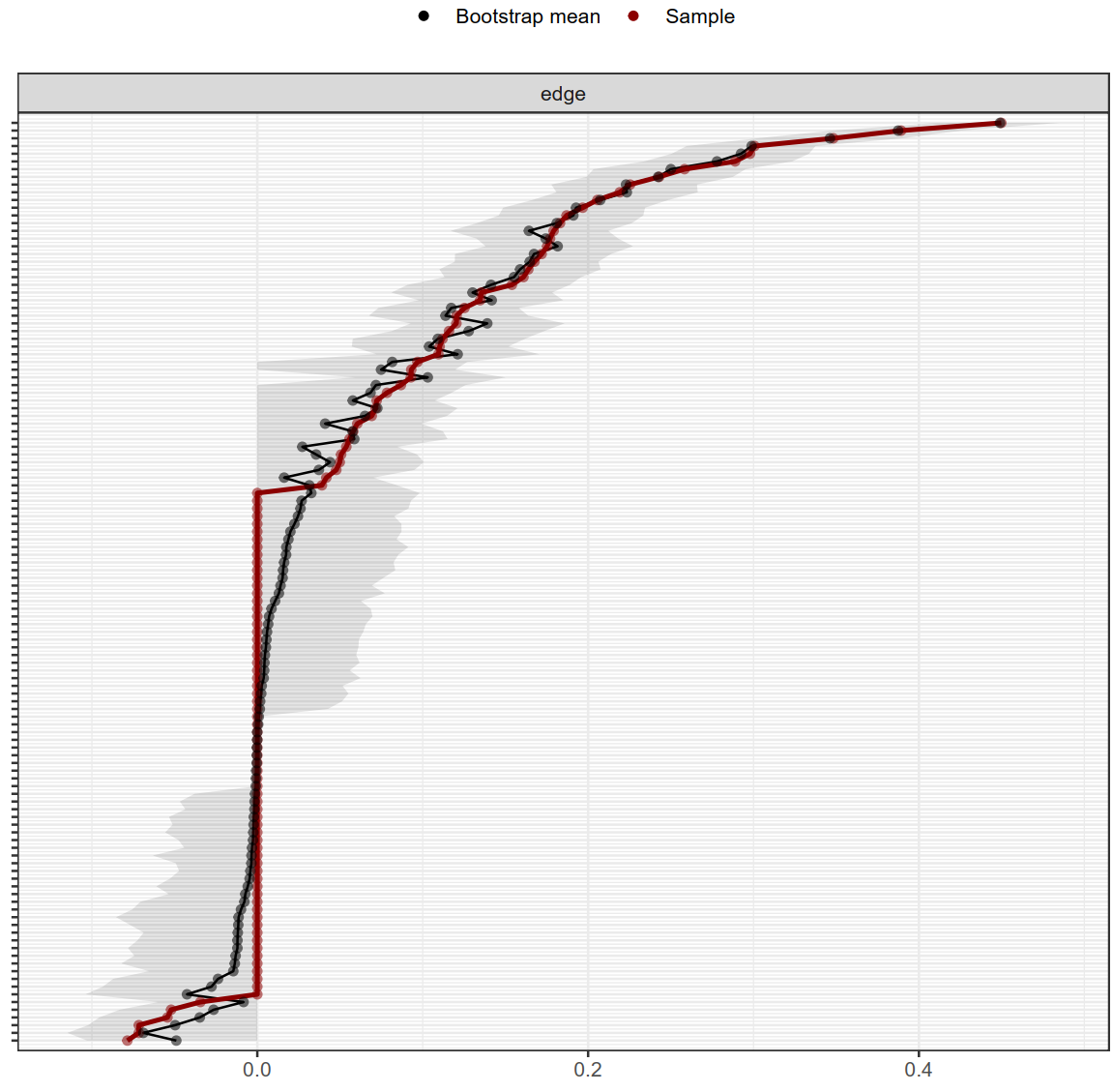


**a)**


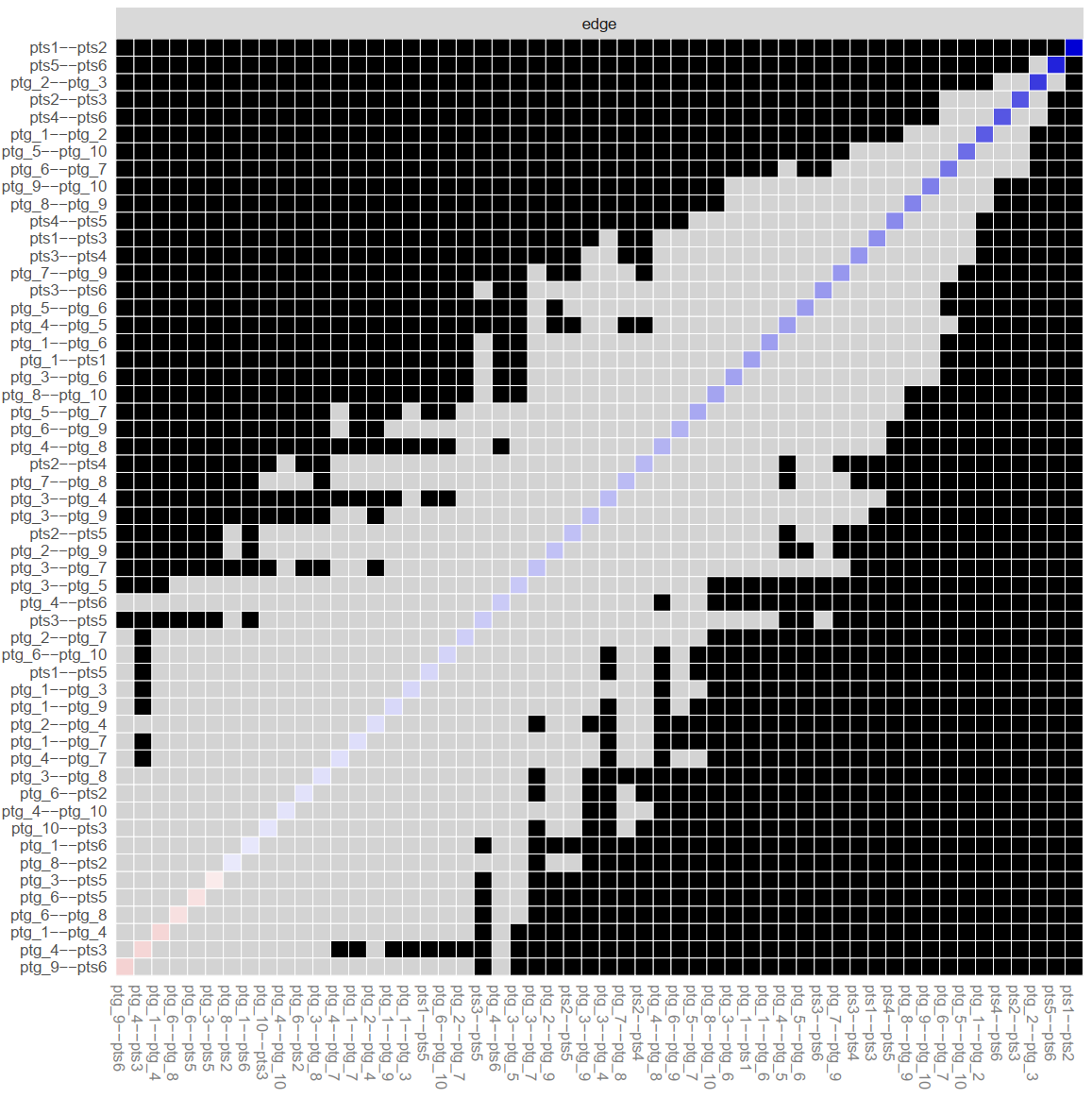


**b)**


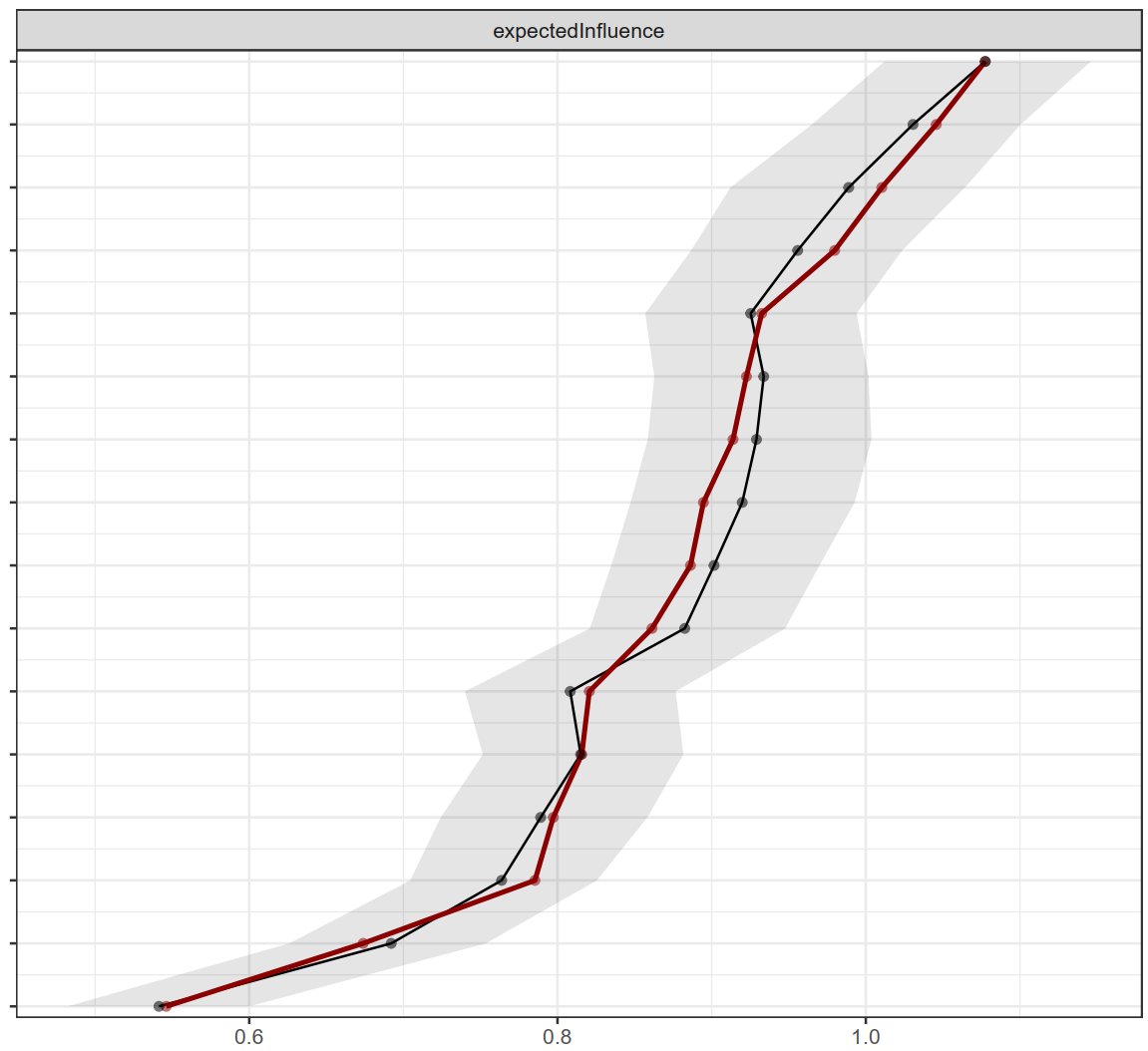


**c)**


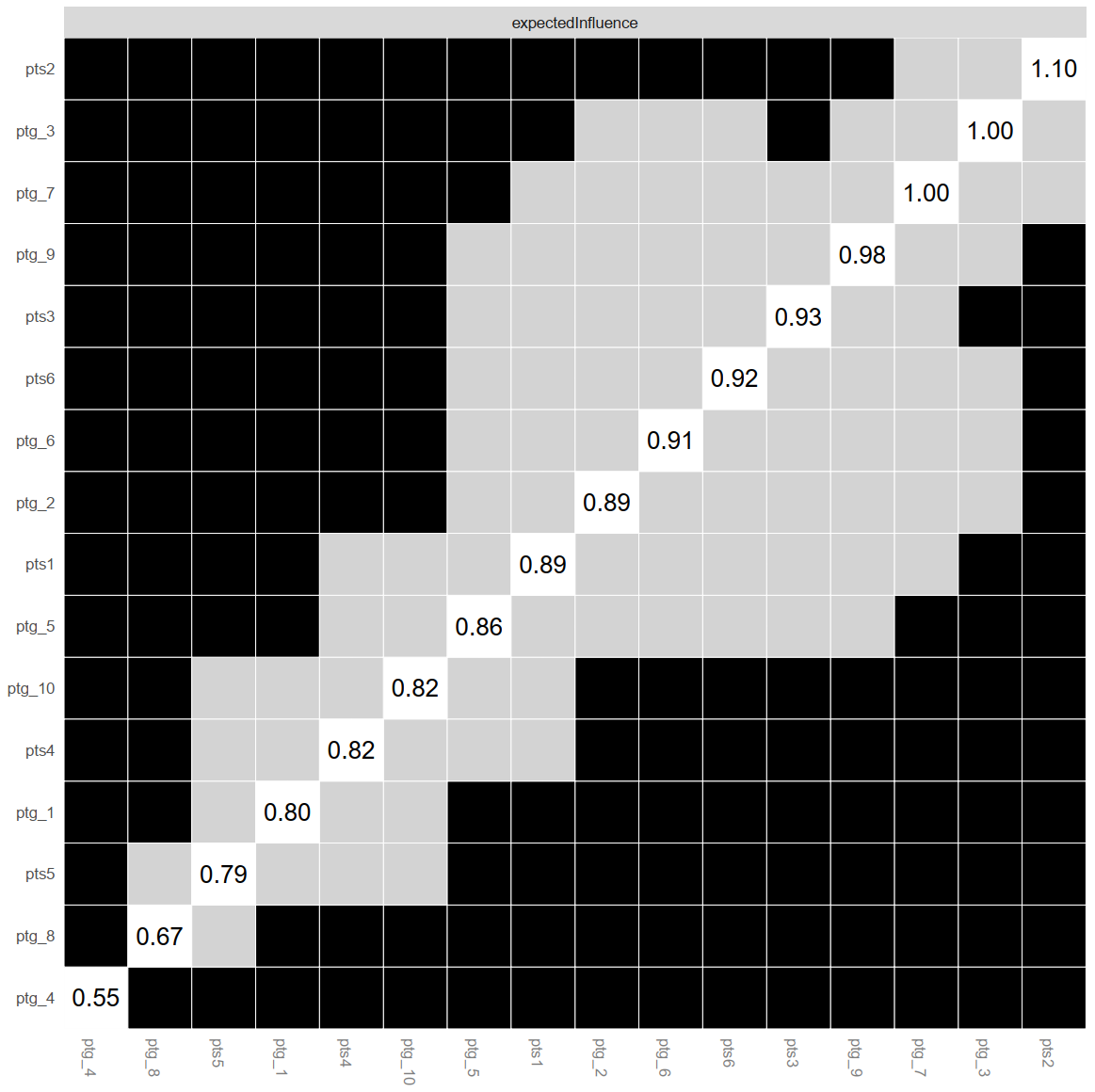


**d)**

**Supplementary Fig 3a-d.** Accuracy of estimated edges and expected influence metrics for network analysis of elements of posttraumatic growth and posttraumatic stress symptoms among Syrian and Iraqi refugees residing in Turkey. a) Estimates and 95% confidence intervals for edge weights based on 3000 bootstrapped samples. b) Results of edge weights difference tests based on 3000 bootstrapped samples. Black squares indicate significant difference in estimate. Only non-zero edge weights shown. c) a) Estimates and 95% confidence intervals for expected influence based on 3000 bootstrapped samples. d) Results of expected influence difference tests based on 3000 bootstrapped samples. Black squares indicate significant difference in estimate.
