# Supplementary material for: Posttraumatic growth and posttraumatic stress – a network analysis among Syrian and Iraqi refugees
Source: Eur J Psychotraumatol. 2022 Sep 21;13(2):2117902. doi: 10.1080/20008066.2022.2117902 (PMC9518504; doi:10.1080/20008066.2022.2117902)
Supplement: Supplemental Material [file ZEPT_A_2117902_SM9037.rtf]

Supplementary Table 1 for Kangaslampi, Peltonen, & Hall (2022). Posttraumatic growth and posttraumatic stress – A network analysis among Syrian and Iraqi refugees 
 
Correlations with confidence intervals between different aspects of posttraumatic growth (PTG), as measured by the PTGI-SF measure, and different posttraumatic stress symptoms (PTSS), as measured by the PCL-C short form measure, as well as total PTG and total PTSS.
 
Variable	M	SD	1	2	3	4	5	6	7	8	9	10	11	12	13	14	15	16	17	
																				
1. PTG-1	3.06	1.37	 	 	 	 	 	 	 	 	 	 	 	 	 	 	 	 	 	
 	 	 	 	 	 	 	 	 	 	 	 	 	 	 	 	 	 	 	 	
2. PTG-2	3.29	1.24	.54**	 	 	 	 	 	 	 	 	 	 	 	 	 	 	 	 	
 	 	 	[.51, .56]	 	 	 	 	 	 	 	 	 	 	 	 	 	 	 	 	
 	 	 	 	 	 	 	 	 	 	 	 	 	 	 	 	 	 	 	 	
3. PTG-3	3.32	1.22	.45**	.63**	 	 	 	 	 	 	 	 	 	 	 	 	 	 	 	
 	 	 	[.42, .47]	[.60, .65]	 	 	 	 	 	 	 	 	 	 	 	 	 	 	 	
 	 	 	 	 	 	 	 	 	 	 	 	 	 	 	 	 	 	 	 	
4. PTG-4	2.98	1.42	.17**	.28**	.35**	 	 	 	 	 	 	 	 	 	 	 	 	 	 	
 	 	 	[.14, .21]	[.25, .32]	[.32, .38]	 	 	 	 	 	 	 	 	 	 	 	 	 	 	
 	 	 	 	 	 	 	 	 	 	 	 	 	 	 	 	 	 	 	 	
5. PTG-5	3.23	1.19	.30**	.40**	.47**	.37**	 	 	 	 	 	 	 	 	 	 	 	 	 	
 	 	 	[.26, .33]	[.37, .43]	[.44, .50]	[.34, .40]	 	 	 	 	 	 	 	 	 	 	 	 	 	
 	 	 	 	 	 	 	 	 	 	 	 	 	 	 	 	 	 	 	 	
6. PTG-6	3.19	1.29	.44**	.47**	.53**	.26**	.49**	 	 	 	 	 	 	 	 	 	 	 	 	
 	 	 	[.41, .47]	[.44, .49]	[.50, .55]	[.23, .29]	[.46, .51]	 	 	 	 	 	 	 	 	 	 	 	 	
 	 	 	 	 	 	 	 	 	 	 	 	 	 	 	 	 	 	 	 	
7. PTG-7	3.32	1.16	.39**	.48**	.54**	.32**	.49**	.57**	 	 	 	 	 	 	 	 	 	 	 	
 	 	 	[.36, .42]	[.45, .51]	[.52, .57]	[.29, .35]	[.47, .52]	[.54, .59]	 	 	 	 	 	 	 	 	 	 	 	
 	 	 	 	 	 	 	 	 	 	 	 	 	 	 	 	 	 	 	 	
8. PTG-8	3.55	1.31	.25**	.33**	.39**	.30**	.37**	.31**	.45**	 	 	 	 	 	 	 	 	 	 	
 	 	 	[.22, .28]	[.30, .36]	[.36, .42]	[.27, .33]	[.34, .40]	[.28, .35]	[.42, .48]	 	 	 	 	 	 	 	 	 	 	
 	 	 	 	 	 	 	 	 	 	 	 	 	 	 	 	 	 	 	 	
9. PTG-9	3.35	1.24	.38**	.46**	.52**	.25**	.43**	.48**	.55**	.52**	 	 	 	 	 	 	 	 	 	
 	 	 	[.35, .41]	[.43, .49]	[.49, .54]	[.21, .28]	[.40, .46]	[.45, .51]	[.52, .57]	[.49, .54]	 	 	 	 	 	 	 	 	 	
 	 	 	 	 	 	 	 	 	 	 	 	 	 	 	 	 	 	 	 	
10. PTG-10	2.98	1.32	.27**	.36**	.37**	.30**	.49**	.39**	.42**	.42**	.51**	 	 	 	 	 	 	 	 	
 	 	 	[.24, .31]	[.32, .39]	[.34, .40]	[.26, .33]	[.47, .52]	[.36, .42]	[.39, .45]	[.39, .45]	[.48, .53]	 	 	 	 	 	 	 	 	
 	 	 	 	 	 	 	 	 	 	 	 	 	 	 	 	 	 	 	 	
11. PTSS-1	3.20	1.12	.34**	.23**	.15**	.06**	.14**	.16**	.15**	.11**	.14**	.16**	 	 	 	 	 	 	 	
 	 	 	[.31, .37]	[.19, .26]	[.11, .18]	[.03, .10]	[.11, .18]	[.13, .20]	[.11, .18]	[.07, .14]	[.10, .17]	[.13, .20]	 	 	 	 	 	 	 	
 	 	 	 	 	 	 	 	 	 	 	 	 	 	 	 	 	 	 	 	
12. PTSS-2	3.06	1.16	.27**	.16**	.14**	.07**	.15**	.15**	.11**	.10**	.08**	.10**	.70**	 	 	 	 	 	 	
 	 	 	[.23, .30]	[.13, .20]	[.10, .17]	[.03, .10]	[.11, .18]	[.12, .19]	[.08, .15]	[.06, .13]	[.05, .12]	[.07, .14]	[.68, .71]	 	 	 	 	 	 	
 	 	 	 	 	 	 	 	 	 	 	 	 	 	 	 	 	 	 	 	
13. PTSS-3	2.76	1.28	.23**	.13**	.08**	.02	.11**	.11**	.08**	.04*	.05**	.11**	.59**	.67**	 	 	 	 	 	
 	 	 	[.19, .26]	[.09, .16]	[.04, .11]	[-.02, .06]	[.07, .15]	[.08, .15]	[.04, .11]	[.01, .08]	[.02, .09]	[.08, .15]	[.56, .61]	[.65, .69]	 	 	 	 	 	
 	 	 	 	 	 	 	 	 	 	 	 	 	 	 	 	 	 	 	 	
14. PTSS-4	2.73	1.35	.16**	.07**	.05*	.11**	.06**	.06**	.03	.02	-.01	.07**	.44**	.53**	.60**	 	 	 	 	
 	 	 	[.13, .20]	[.03, .11]	[.01, .08]	[.07, .14]	[.02, .09]	[.03, .10]	[-.00, .07]	[-.01, .06]	[-.05, .03]	[.04, .11]	[.41, .47]	[.51, .56]	[.57, .62]	 	 	 	 	
 	 	 	 	 	 	 	 	 	 	 	 	 	 	 	 	 	 	 	 	
15. PTSS-5	2.76	1.22	.12**	.07**	.01	.04*	.02	.01	.00	.01	-.03	-.00	.44**	.52**	.55**	.60**	 	 	 	
 	 	 	[.09, .16]	[.03, .11]	[-.02, .05]	[.00, .07]	[-.02, .06]	[-.02, .05]	[-.04, .04]	[-.03, .05]	[-.07, .00]	[-.04, .03]	[.41, .47]	[.49, .54]	[.53, .58]	[.57, .62]	 	 	 	
 	 	 	 	 	 	 	 	 	 	 	 	 	 	 	 	 	 	 	 	
16. PTSS-6	2.65	1.32	.17**	.10**	.04*	.11**	.05**	.08**	.05*	.03	-.01	.08**	.43**	.51**	.58**	.65**	.66**	 	 	
 	 	 	[.13, .20]	[.06, .13]	[.01, .08]	[.08, .15]	[.02, .09]	[.04, .11]	[.01, .08]	[-.01, .07]	[-.04, .03]	[.04, .11]	[.40, .46]	[.48, .53]	[.55, .60]	[.63, .67]	[.64, .68]	 	 	
 	 	 	 	 	 	 	 	 	 	 	 	 	 	 	 	 	 	 	 	
17. PTG-total	32.32	8.65	.61**	.72**	.76**	.54**	.70**	.72**	.75**	.64**	.74**	.66**	.23**	.19**	.14**	.09**	.04	.11**	 	
 	 	 	[.59, .63]	[.70, .73]	[.74, .78]	[.51, .56]	[.68, .72]	[.70, .74]	[.74, .77]	[.61, .66]	[.72, .76]	[.64, .68]	[.20, .27]	[.16, .23]	[.10, .18]	[.06, .13]	[-.00, .07]	[.07, .14]	 	
 	 	 	 	 	 	 	 	 	 	 	 	 	 	 	 	 	 	 	 	
18. PTSS-total	17.17	5.93	.26**	.15**	.09**	.08**	.11**	.12**	.08**	.06**	.04*	.11**	.73**	.81**	.83**	.81**	.79**	.81**	.16**	
 	 	 	[.22, .29]	[.11, .18]	[.05, .13]	[.05, .12]	[.07, .14]	[.08, .15]	[.05, .12]	[.02, .10]	[.00, .08]	[.07, .14]	[.72, .75]	[.79, .82]	[.82, .84]	[.80, .82]	[.78, .80]	[.80, .82]	[.13, .20]	
 	 	 	 	 	 	 	 	 	 	 	 	 	 	 	 	 	 	 	 	

Note. M and SD are used to represent mean and standard deviation, respectively. Values in square brackets indicate the 95% confidence interval for each correlation. * indicates p < .05. ** indicates p < .01. See Table 2 for order and meaning of PTG and PTSS items.
