# Supplementary material for: Posttraumatic growth and posttraumatic stress – a network analysis among Syrian and Iraqi refugees
Source: Eur J Psychotraumatol. 2022 Sep 21;13(2):2117902. doi: 10.1080/20008066.2022.2117902 (PMC9518504; doi:10.1080/20008066.2022.2117902)
Supplement: Supplemental Material [file ZEPT_A_2117902_SM9030.docx]

**Supplementary Information for**

**Kangaslampi, Peltonen, & Hall (2022). Posttraumatic growth and posttraumatic stress – A network analysis among Syrian and Iraqi refugees**

**Detailed description of research setting and procedure**

*Civil wars in Iraq and Syria*

The armed conflicts in Iraq and Syria are two of the most destructive wars taking place in the world today. Both countries have witnessed the majority of the world’s battle-related deaths since 2011 (Pettersson and Eck 2018) which has generated massive dislocation of civilian populations. Syria, where much of the violence has taken place, has seen more than half of its civilian population displaced. 5.6 million Syrian refugees now reside in other countries and a further 6.6 million remain internally displaced (UNHCR 2018). The effects of these conflicts are felt around the world due to the refugee flows originating from these conflict zones. Studying refugees from these conflicts is important due to their unique tragic experiences during the war, their integration into host societies and future role in rebuilding their homelands.

The situation in both countries was characterized by the systematic use of indiscriminate violence during the study period, including the widespread use of shelling and bombing of entire neighborhoods and towns. Combat between Sunni Islamist and Syrian and Iraqi government forces constituted the main axis of fighting in the region. With the support of regional power Iran and allied militias, the armed forces of Shia-backed governments in both Syria and Iraq battled the Islamic State, which claimed to represent the interests of all Sunni Muslims.

*Community-based sampling*

To study the network dynamics between PTSS and PTG in a war exposed refugee sample, reaching populations directly affected by war is of central importance. Community-based samples of refugees with a high degree of exposure to potentially traumatic events are rarely studied due to the inherent difficulty in gaining access to such populations and the logistical barriers to data collection. We thus designed our sampling approach to ensure we could access a large and diverse community-based sample in Turkey.

*Procedure*

In order to gain access to this population, a team of local assistants, themselves refugees from Syria and Iraq, were recruited and trained to administer the study. The use of local assistants in the administration of the study helped to ensure that the study was carried out with cultural sensitivity and in a context of interpersonal trust. We acknowledge the important contribution and immense work effort invested by these local research assistants, without whom the research would not have been possible to carry out.

The original translation of the research materials was conducted by a professional translator. During the training period, this original Arabic translation of the instructions, experiment and survey was evaluated by our field research team and adapted so as to reflect the Arabic dialects spoken in the local community. The new draft was then again evaluated and approved by the original translator (the new version was equally correct but more colloquial in nature). The standardized Arabic translations of the civilian version of the PTSD Checklist (PCL-C) and Posttraumatic Growth Inventory-Short Form (PTGI-SF) were left unchanged during this process.

Once the materials were ready for testing, we recruited a focus group of eight refugees who agreed to act as pilot participants. Under the supervision of the fieldwork coordinator, the assistants practiced conducting the study multiple times over several evenings with this pilot group. The group was also invited to provide feedback, which resulted in an additional round of improvements addressing the clarity of the instructions for the participants and some question wording. After a week of testing, the team and the instruments were ready for the field.

Building on trust networks established through the fieldwork, we opted for community-based sampling, whereby existing participants recruit future participants from among their social networks, a sampling procedure often used to identify otherwise hidden populations. The team was trained in the city of Konya and then distributed to other cities depending on their personal networks and knowledge of local neighborhoods. In each location, each team of two to three assistants first established relationships of trust with individuals and families that were well-known and well-regarded in the local community. Upon completion of the session, the participants were asked to put us in contact with additional families for future sessions. Much of this work occurred in the evenings after work. During the day, we worked to supplement this strategy and further diversify the sample by approaching refugees standing in breadlines, outside aid organizations, in public transportation hubs, at universities and in front of refugee camps.

Data collection occurred in three waves: 2016, 2017, and 2019. The same procedure for participant recruitment was carried in eleven cities in total: Adana, Ankara, Antalya, Antakya, Balıkesir, Eskişehir, Istanbul, Kahramanmaraş, Konya, Mersin and Yalova. Figure 1 displays our fieldwork locations.

**
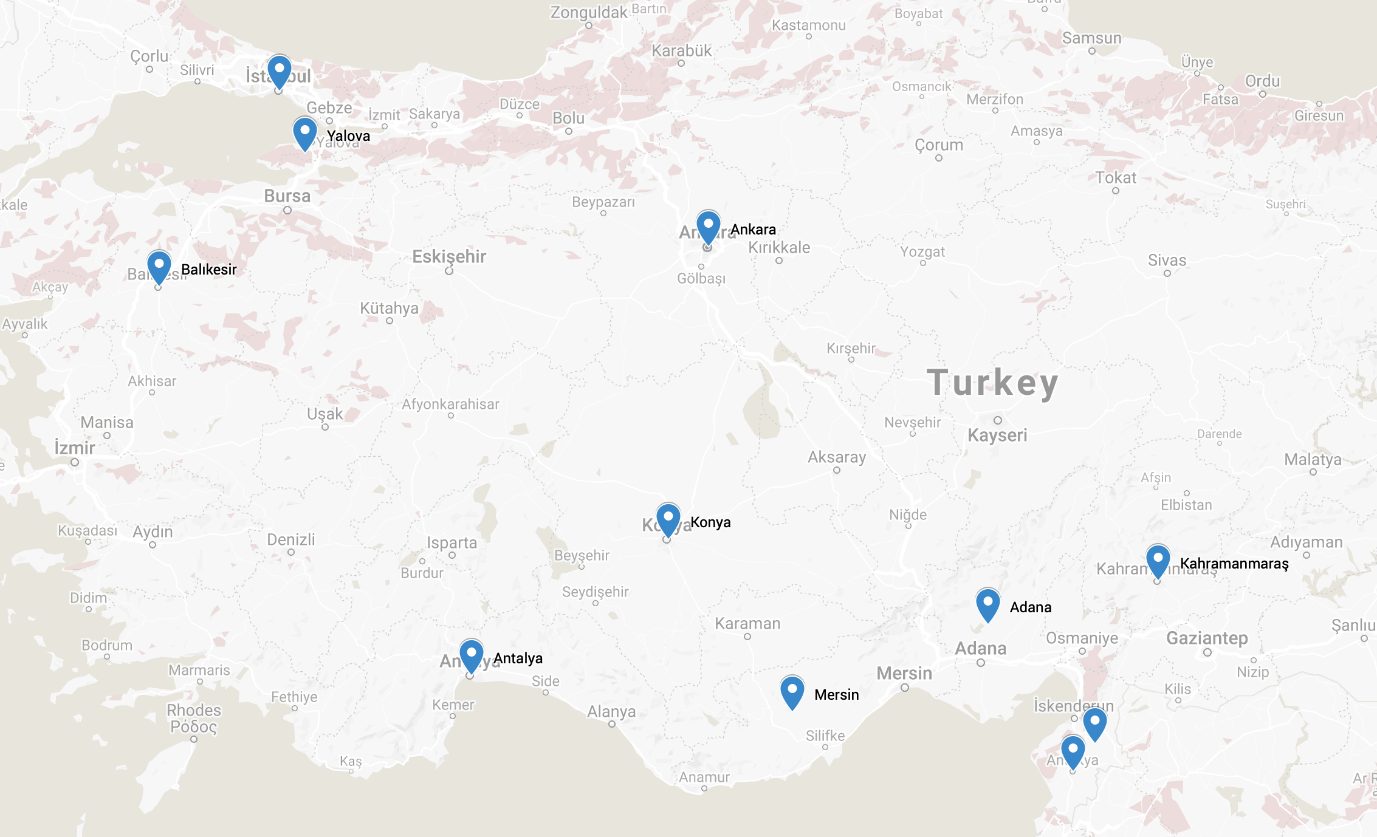
**

*Figure 1*. Map of fieldwork locations

Potential participants were informed that this was an Uppsala University-based study. Those approached were also provided with the contact information of the third author. The fact that the study was conducted by a Swedish university further facilitated recruitment, as Sweden has a positive reputation as a good-faith actor in the Middle Eastern context. Before being asked to give consent to participate, those approached were informed that participation was completely voluntary and they could discontinue their involvement at any time and for any reason. Those who elected to participate received a small sum of money – 10 Turkish lira during the 2016 wave and 20 Turkish lira during the 2017 and 2019 waves – as compensation for their time. The study was administered inside the homes of families on tablet computers individually and anonymously. The only exception was made for illiterate participants. In these cases, the research assistant conducted an oral interview. Female RAs interviewed female respondents. This project received approval from the Uppsala University Ethical Review Board.

*References*

Pettersson, T., & Eck, K. (2018). Organized violence, 1989–2017. Journal of Peace Research, 55(4), 535–547.

UNHCR. (2018). Provision of Life-Saving Assistance and Supporting Communities: End of Year Report 2018. United Nations.
