# Supplementary material for: Posttraumatic growth and posttraumatic stress – a network analysis among Syrian and Iraqi refugees
Source: Eur J Psychotraumatol. 2022 Sep 21;13(2):2117902. doi: 10.1080/20008066.2022.2117902 (PMC9518504; doi:10.1080/20008066.2022.2117902)
Supplement: Supplemental Material [file ZEPT_A_2117902_SM9029.docx]

**Supplementary statistics and analyses for
Kangaslampi, Peltonen, & Hall (2022). Posttraumatic growth and posttraumatic stress – A network analysis among Syrian and Iraqi refugees**

Contents

1. Full results of regression model of total PTSS and squared total PTSS predicting total posttraumatic growth
2. Graphical representation of node strength and node expected influence for PTG+PTSS network
3. Regularized networks with EBICglasso
4. PTG + PTSS networks without Changed Priorities item
5. All edge strength estimates
6. Raw and scaled centrality estimates
7. Full results of regression model of total PTSS and squared total PTSS predicting total posttraumatic growth

Estimate *SE* *t* *p*

Intercept 36.42 1.25 29.06 < .0001

Total PTSS Squared 0.03 0.004 6.92 < .0001

Total PTSS -0.79 0.15 -5.23 < .00001

Residual standard error: 8.35 on 2858 degrees of freedom

Multiple *R*^2^ = .042 Adjusted *R*^2^ = .042

*F*(2,2858) = 63.12, *p* < .00001

1. Graphical representation of node strength and node expected influence for PTG+PTSS network


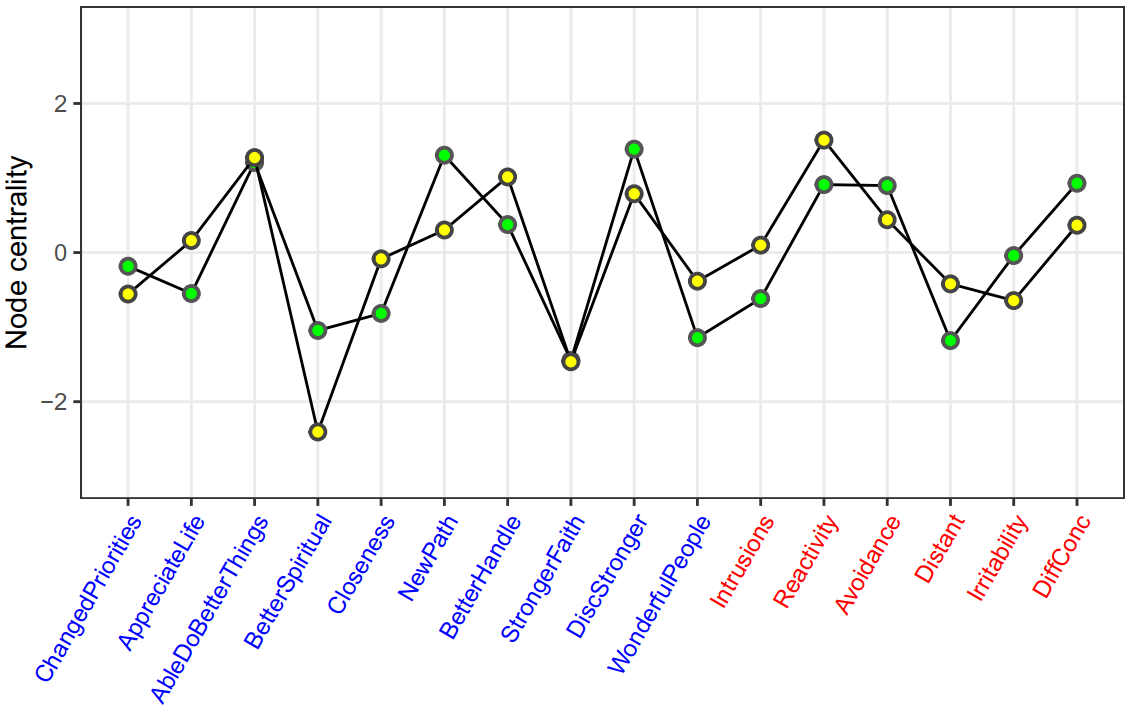


**Supplementary Fig 5.** Node strength (green) and 1-step node expected influence (yellow) for network of aspects of posttraumatic growth (presented in blue) and types of posttraumatic stress symptoms (presented in red) among Syrian and Iraqi refugees residing in Turkey. Scaled values presented.

1. Regularized networks with EBICglasso

For purposes of comparison with earlier published research, and as sensitivity analyses, we also estimated the PTG and PTG+PTSS networks using the EBICglasso function from the *qgraph* R package. Here, we used the extended Bayesian information criterion with hypertuning parameter γ set to 0.5 to select the tuning parameter λ.

3.1 PTG only network with EBICglasso


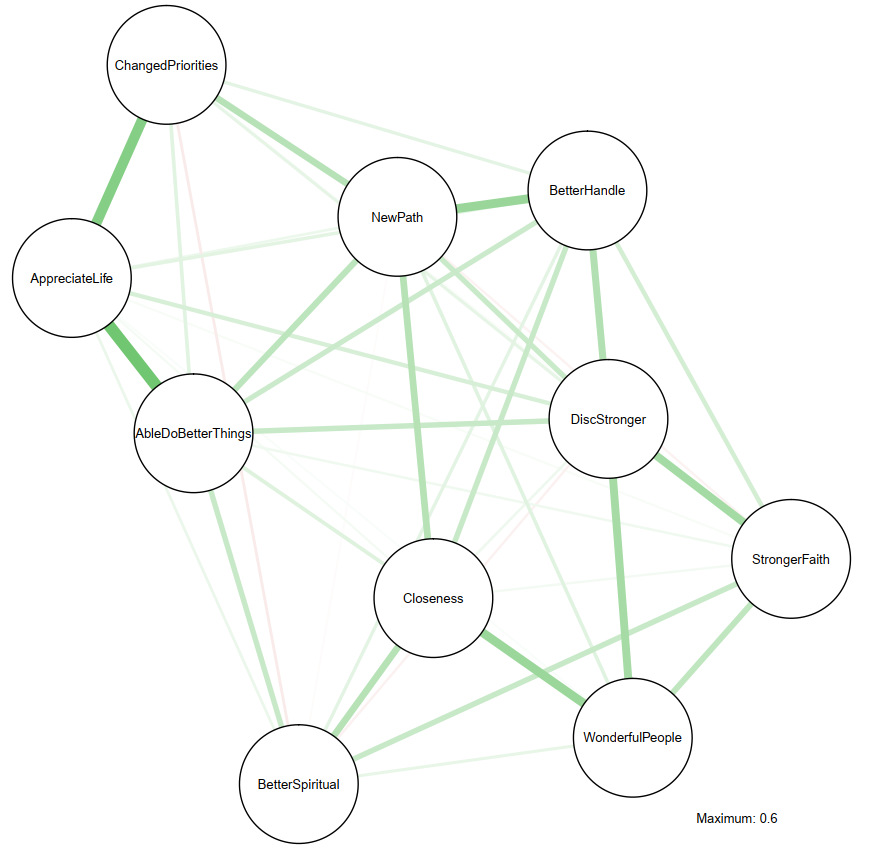


**Supplementary Fig 6.** Gaussian graphical model of aspects of posttraumatic growth for Syrian and Iraqi refugees residing in Turkey. Edge thickness represents degree of association as partial correlations, green edges indicate positive association, and red edges indicate negative association. The EBICglasso algorithm used for estimating the network and the *spring* algorithm used for layout determination.


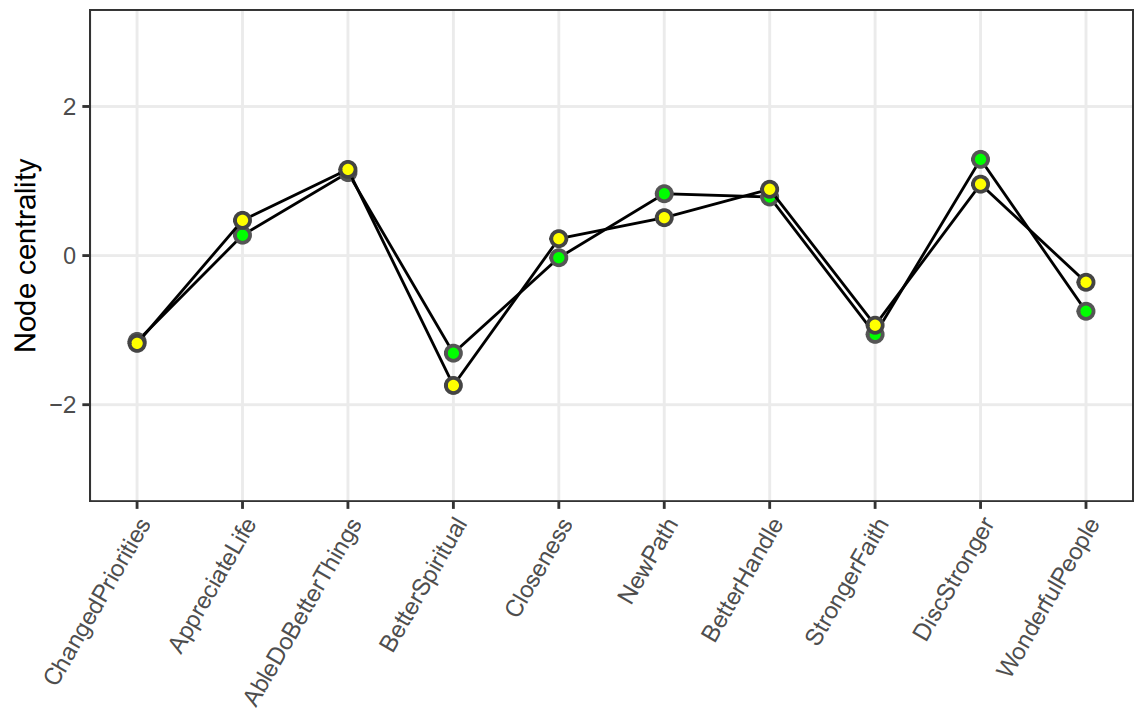


**Supplementary Fig 7.** Node strength (green) and 1-step expected influence (yellow) as measures of node centrality for network of aspects of posttraumatic growth, estimated using the EBICglasso algorithm. Scaled values presented.

Correlation between edge weights in network estimated using the ggmModSelect algorithm and network estimated with EBICglasso was *r* = .98, 95% CI [.96, .99], indicating highly similar structures.

3.2 PTG+PTSS network with EBICglasso


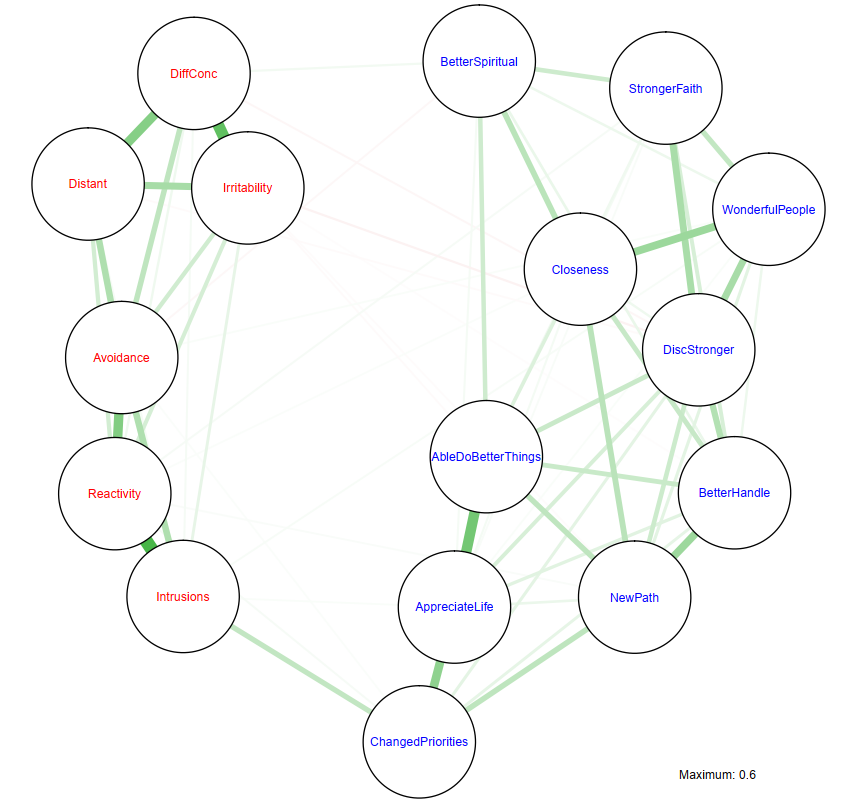


**Supplementary Fig 8.** Gaussian graphical model of aspects of posttraumatic growth and types of posttraumatic stress symptoms for Syrian and Iraqi refugees residing in Turkey. Edge thickness represents degree of association as partial correlations, green edges indicate positive association, and red edges indicate negative association. Posttraumatic growth items presented in blue, posttraumatic stress symptom items presented in red. The EBICglasso algorithm used for estimating the network and the *spring* algorithm used for layout determination.


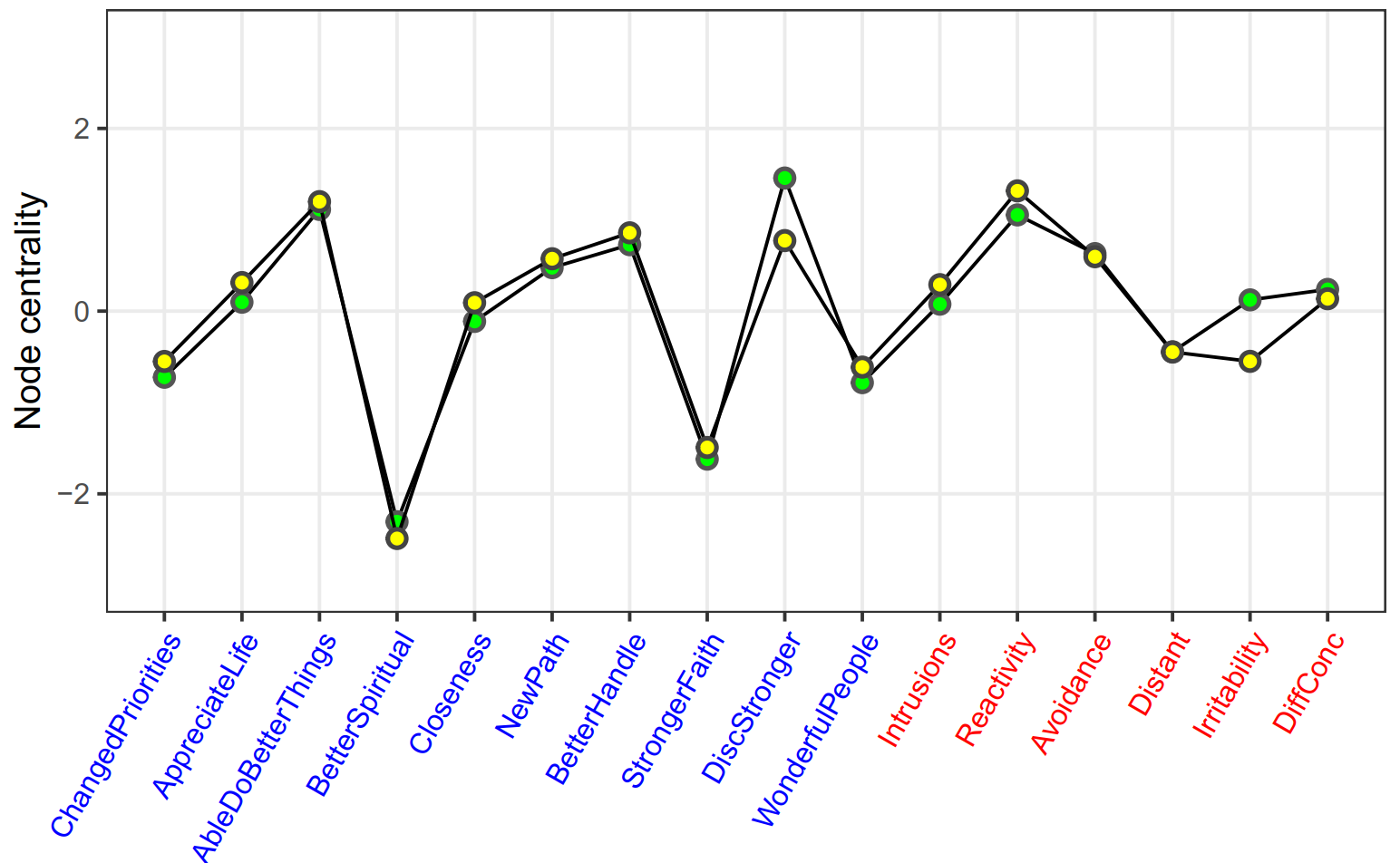


**Supplementary Fig 9.** Node strength (green) and 1-step expected influence (yellow) as measures of node centrality for network of aspects of posttraumatic growth (presented in blue) and types of posttraumatic stress symptoms (presented in red), estimated using the EBICglasso algorithm. Scaled values presented.


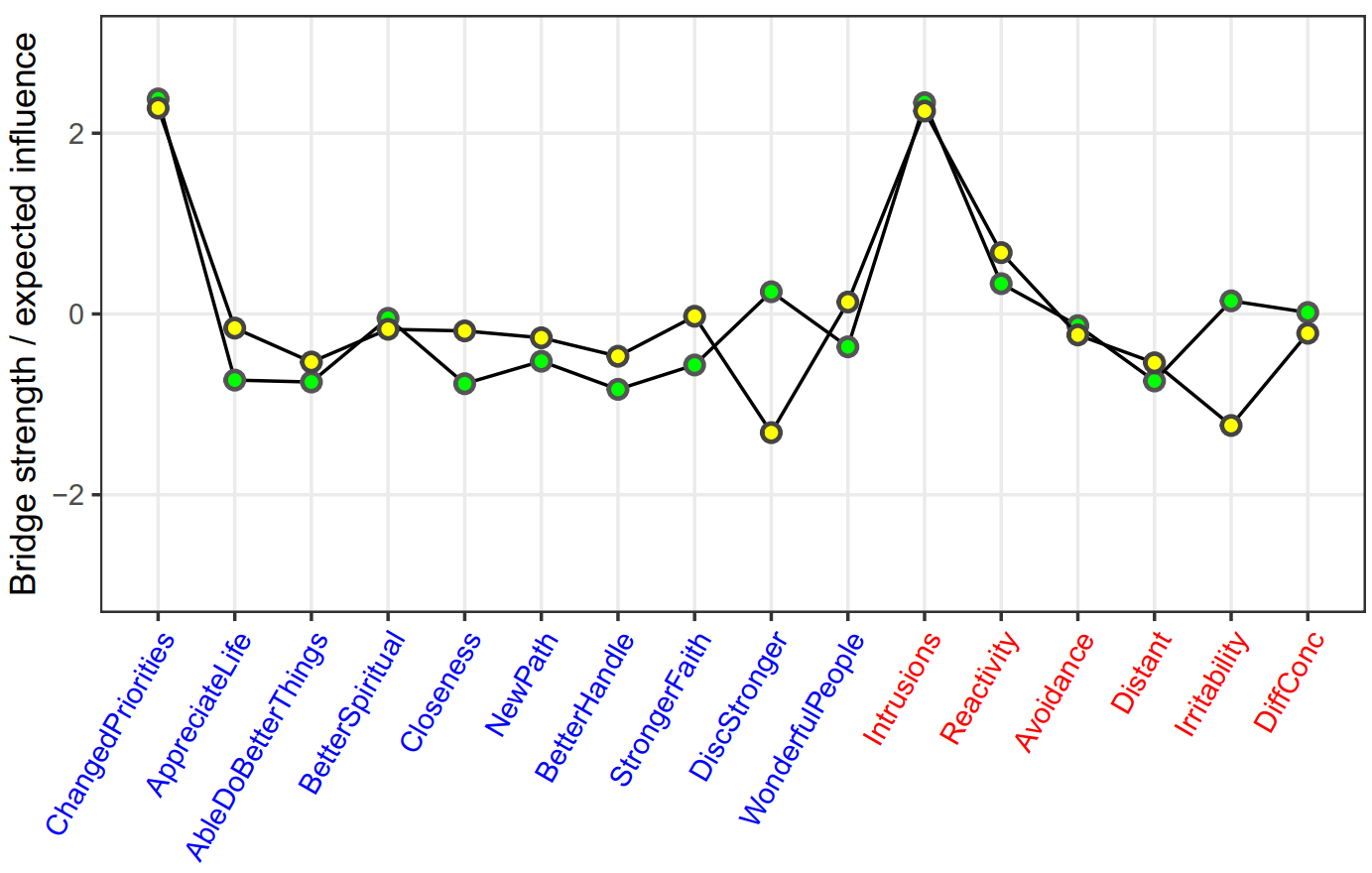


**Supplementary Fig 10.** Bridge strength (green) and 1-step bridge expected influence (yellow) for network of aspects of posttraumatic growth (presented in blue) and types of posttraumatic stress symptoms (presented in red), estimated using the EBICglasso algorithm. Scaled values presented.

Correlation between edge weights in network estimated using the ggmModSelect algorithm and network estimated with EBICglasso was *r* = .98, 95% CI [.97, .99], indicating highly similar structures.

1. PTG + PTSS networks without Changed Priorities item


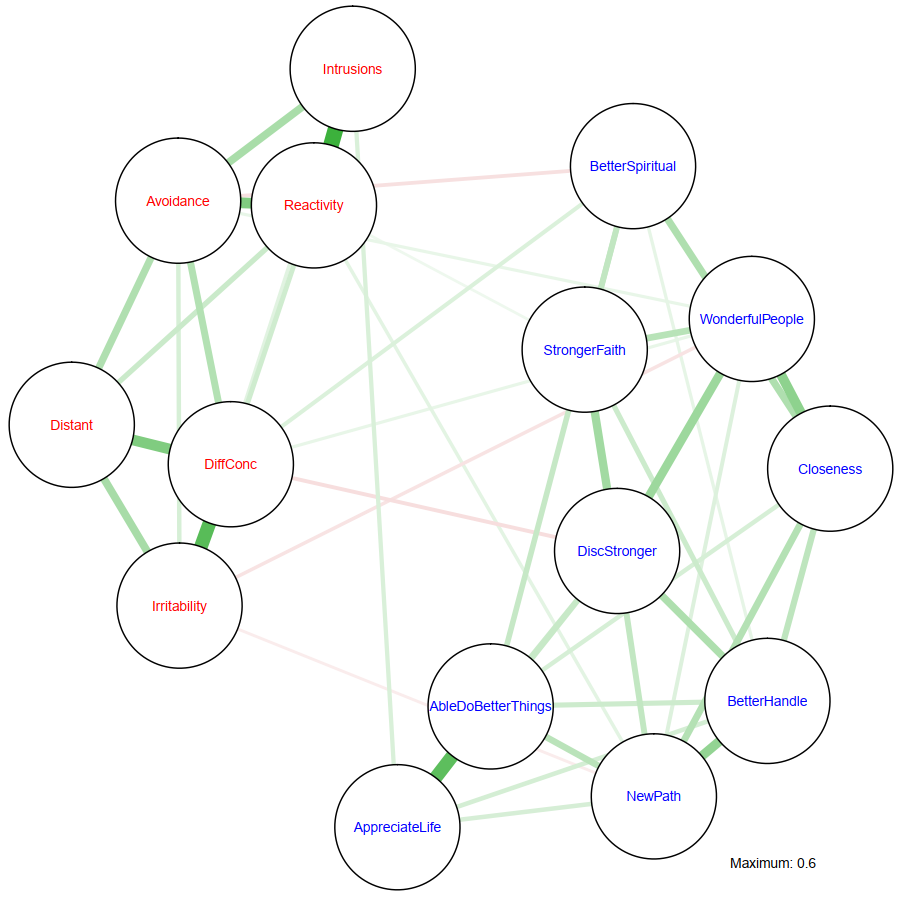


**Supplementary Fig 11.** Gaussian graphical model of aspects of posttraumatic growth and types of posttraumatic stress symptoms excluding the Changed Priorities item, for Syrian and Iraqi refugees residing in Turkey. Edge thickness represents degree of association as partial correlations, green edges indicate positive association, and red edges indicate negative association. Posttraumatic growth items presented in blue, posttraumatic stress symptom items presented in red. The ggmModSelect algorithm used for estimating the network and the *spring* algorithm used for layout determination.


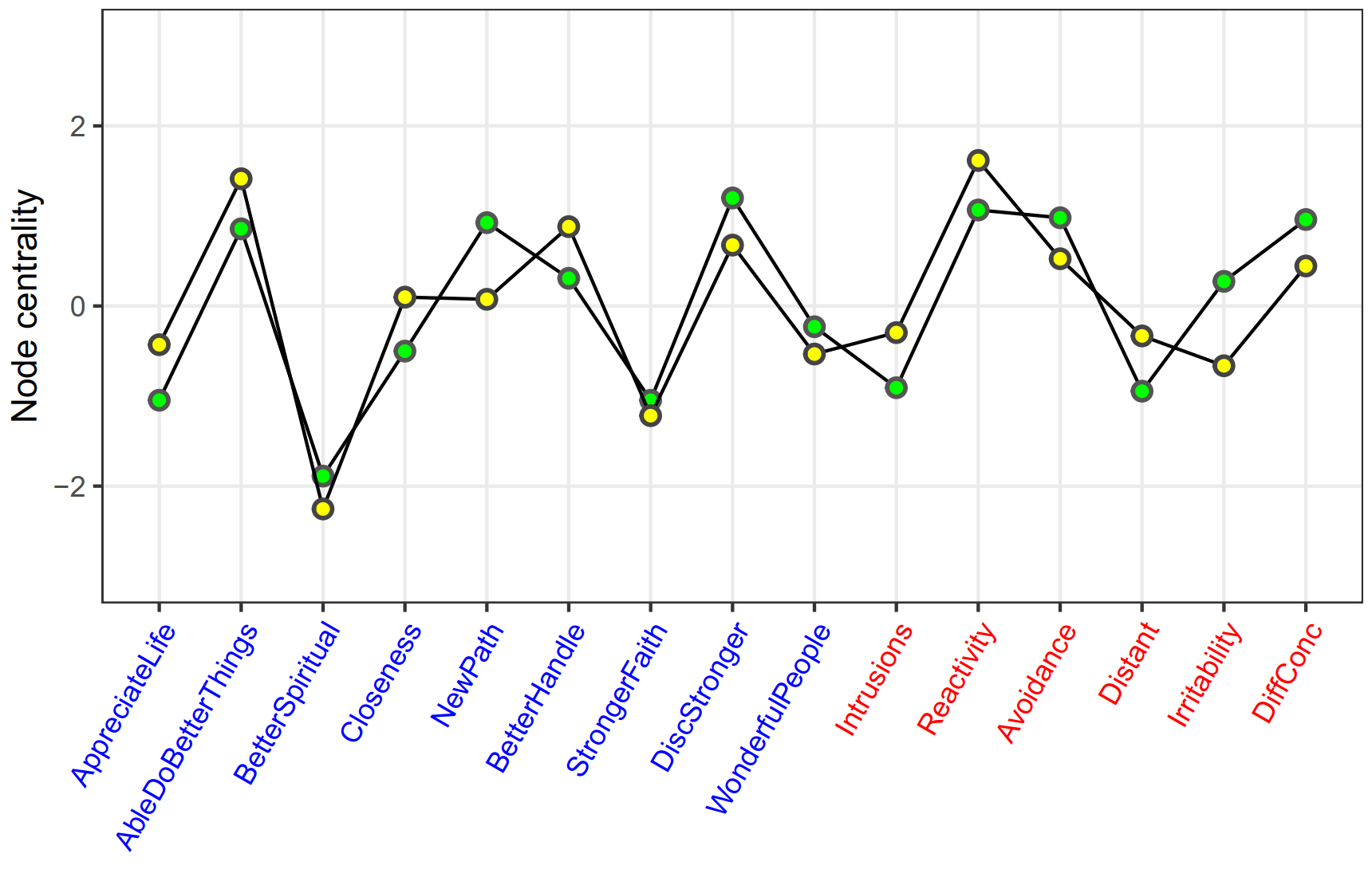


**Supplementary Fig 12.** Node strength (green) and 1-step expected influence (yellow) as measures of node centrality for network of aspects of posttraumatic growth (presented in blue) and types of posttraumatic stress symptoms (presented in red), excluding the Changed Priorities item, estimated using the ggmModSelect algorithm. Scaled values presented.


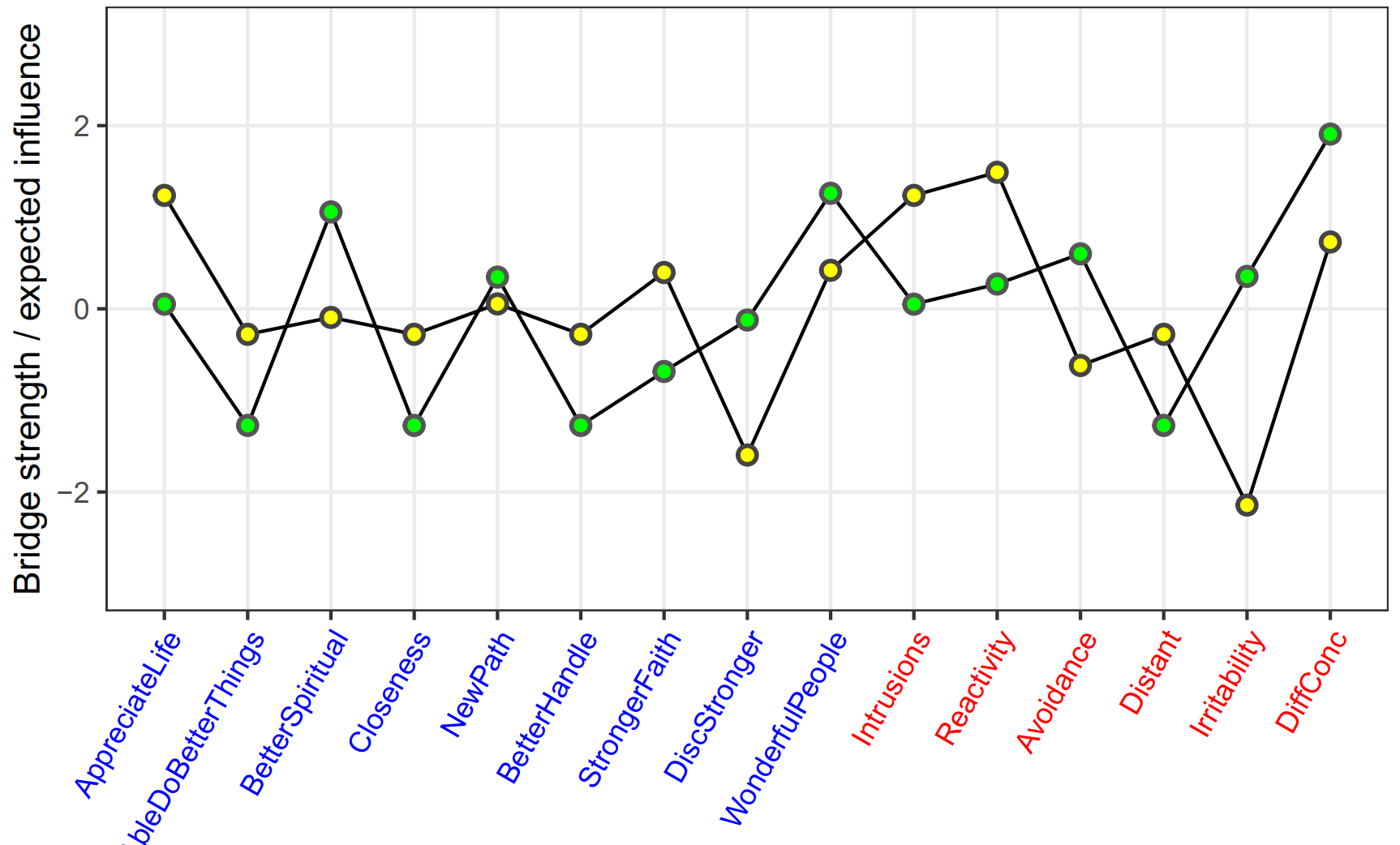


**Supplementary Fig 13.** Bridge strength (green) and 1-step bridge expected influence (yellow) for network of aspects of posttraumatic growth (presented in blue) and types of posttraumatic stress symptoms (presented in red), excluding the Changed Priorities item, estimated using the ggmModSelect algorithm. Scaled values presented.

1. All edge weight estimates

All non-zero edge weight estimates, in order of absolute value, are presented below for all estimated networks. Negative edge weight estimates presented in red.

5.1. Edge strength estimates for PTG only network using ggmModSelect algorithm

AppreciateLife –– AbleDoBetterThings 0.349

ChangedPriorities –– AppreciateLife 0.3

Closeness –– WonderfulPeople 0.269

NewPath –– BetterHandle 0.237

DiscStronger –– WonderfulPeople 0.229

StrongerFaith –– DiscStronger 0.218

BetterSpiritual –– Closeness 0.186

ChangedPriorities –– NewPath 0.184

BetterHandle –– DiscStronger 0.183

Closeness –– NewPath 0.172

StrongerFaith –– WonderfulPeople 0.168

AbleDoBetterThings –– NewPath 0.163

Closeness –– BetterHandle 0.153

BetterSpiritual –– StrongerFaith 0.149

AbleDoBetterThings –– DiscStronger 0.133

AbleDoBetterThings –– BetterSpiritual 0.126

NewPath –– DiscStronger 0.122

BetterHandle –– StrongerFaith 0.119

AbleDoBetterThings –– BetterHandle 0.117

AppreciateLife –– DiscStronger 0.111

AbleDoBetterThings –– Closeness 0.099

AppreciateLife –– BetterHandle 0.087

NewPath –– WonderfulPeople 0.08

ChangedPriorities –– BetterSpiritual -0.07

ChangedPriorities –– AbleDoBetterThings 0.068

ChangedPriorities –– BetterHandle 0.063

AppreciateLife –– BetterSpiritual 0.061

ChangedPriorities –– DiscStronger 0.06

BetterSpiritual –– BetterHandle 0.054

5.2 Edge strength estimates for PTG+PTSS network using ggmModSelect algorithm

Intrusions –– Reactivity 0.45

Irritability –– DiffConc 0.389

AppreciateLife –– AbleDoBetterThings 0.348

Reactivity –– Avoidance 0.301

Distant –– DiffConc 0.298

ChangedPriorities –– AppreciateLife 0.289

Closeness –– WonderfulPeople 0.258

NewPath –– BetterHandle 0.243

DiscStronger –– WonderfulPeople 0.225

StrongerFaith –– DiscStronger 0.219

Distant –– Irritability 0.206

Intrusions –– Avoidance 0.197

Avoidance –– Distant 0.187

BetterHandle –– DiscStronger 0.183

Avoidance –– DiffConc 0.179

Closeness –– NewPath 0.177

BetterSpiritual –– Closeness 0.175

ChangedPriorities –– NewPath 0.172

ChangedPriorities –– Intrusions 0.168

AbleDoBetterThings –– NewPath 0.164

StrongerFaith –– WonderfulPeople 0.161

Closeness –– BetterHandle 0.154

NewPath –– DiscStronger 0.135

BetterSpiritual –– StrongerFaith 0.135

Reactivity –– Distant 0.125

BetterHandle –– StrongerFaith 0.12

AbleDoBetterThings –– BetterSpiritual 0.12

AbleDoBetterThings –– DiscStronger 0.116

Reactivity –– Irritability 0.112

AppreciateLife –– DiscStronger 0.11

AbleDoBetterThings –– BetterHandle 0.109

AbleDoBetterThings –– Closeness 0.097

BetterSpiritual –– DiffConc 0.093

Avoidance –– Irritability 0.093

AppreciateLife –– BetterHandle 0.087

DiscStronger –– DiffConc -0.079

NewPath –– WonderfulPeople 0.078

Intrusions –– Irritability 0.072

BetterSpiritual –– Avoidance -0.072

ChangedPriorities –– BetterSpiritual -0.072

ChangedPriorities –– AbleDoBetterThings 0.072

ChangedPriorities –– DiscStronger 0.069

AppreciateLife –– BetterSpiritual 0.06

ChangedPriorities –– BetterHandle 0.058

BetterSpiritual –– BetterHandle 0.056

NewPath –– StrongerFaith -0.054

AbleDoBetterThings –– StrongerFaith 0.054

NewPath –– Irritability -0.052

NewPath –– Reactivity 0.051

BetterSpiritual –– WonderfulPeople 0.05

WonderfulPeople –– Avoidance 0.048

ChangedPriorities –– DiffConc 0.042

StrongerFaith –– Reactivity 0.039

AbleDoBetterThings –– Irritability -0.034

5.3 Edge weight estimates for PTG only network using EBICglasso algorithm

AppreciateLife –– AbleDoBetterThings 0.335

ChangedPriorities –– AppreciateLife 0.287

NewPath –– BetterHandle 0.239

Closeness –– WonderfulPeople 0.236

StrongerFaith –– DiscStronger 0.214

DiscStronger –– WonderfulPeople 0.209

BetterHandle –– DiscStronger 0.181

BetterSpiritual –– Closeness 0.172

Closeness –– NewPath 0.171

ChangedPriorities –– NewPath 0.168

AbleDoBetterThings –– NewPath 0.154

StrongerFaith –– WonderfulPeople 0.149

BetterSpiritual –– StrongerFaith 0.132

Closeness –– BetterHandle 0.131

AbleDoBetterThings –– DiscStronger 0.131

AbleDoBetterThings –– BetterSpiritual 0.129

NewPath –– DiscStronger 0.126

AbleDoBetterThings –– BetterHandle 0.122

BetterHandle –– StrongerFaith 0.102

AppreciateLife –– DiscStronger 0.093

AbleDoBetterThings –– Closeness 0.078

NewPath –– WonderfulPeople 0.072

ChangedPriorities –– AbleDoBetterThings 0.071

AppreciateLife –– BetterHandle 0.067

ChangedPriorities –– BetterHandle 0.064

BetterSpiritual –– BetterHandle 0.064

ChangedPriorities –– DiscStronger 0.059

BetterSpiritual –– WonderfulPeople 0.053

ChangedPriorities –– BetterSpiritual -0.047

AppreciateLife –– BetterSpiritual 0.045

AppreciateLife –– NewPath 0.043

Closeness –– DiscStronger 0.039

BetterHandle –– WonderfulPeople 0.036

AbleDoBetterThings –– StrongerFaith 0.036

BetterSpiritual –– DiscStronger -0.033

NewPath –– StrongerFaith -0.031

Closeness –– StrongerFaith 0.029

AppreciateLife –– Closeness 0.025

AppreciateLife –– StrongerFaith 0.018

AppreciateLife –– WonderfulPeople 0.014

ChangedPriorities –– StrongerFaith 0.012

BetterSpiritual –– NewPath -0.009

5.4 Edge weight estimates for PTG+PTSS network using EBICglasso algorithm

Intrusions –– Reactivity 0.43

Irritability –– DiffConc 0.372

AppreciateLife –– AbleDoBetterThings 0.33

Reactivity –– Avoidance 0.293

Distant –– DiffConc 0.285

ChangedPriorities –– AppreciateLife 0.264

Closeness –– WonderfulPeople 0.232

NewPath –– BetterHandle 0.231

Distant –– Irritability 0.207

DiscStronger –– WonderfulPeople 0.205

StrongerFaith –– DiscStronger 0.202

Intrusions –– Avoidance 0.189

Avoidance –– Distant 0.188

BetterHandle –– DiscStronger 0.178

Closeness –– NewPath 0.163

BetterSpiritual –– Closeness 0.161

ChangedPriorities –– NewPath 0.154

Avoidance –– DiffConc 0.151

AbleDoBetterThings –– NewPath 0.151

ChangedPriorities –– Intrusions 0.145

StrongerFaith –– WonderfulPeople 0.143

Closeness –– BetterHandle 0.133

AbleDoBetterThings –– DiscStronger 0.127

AbleDoBetterThings –– BetterHandle 0.126

NewPath –– DiscStronger 0.12

BetterSpiritual –– StrongerFaith 0.119

AbleDoBetterThings –– BetterSpiritual 0.115

Avoidance –– Irritability 0.11

Reactivity –– Distant 0.107

Reactivity –– Irritability 0.099

BetterHandle –– StrongerFaith 0.095

AppreciateLife –– DiscStronger 0.095

AbleDoBetterThings –– Closeness 0.081

AppreciateLife –– BetterHandle 0.071

ChangedPriorities –– AbleDoBetterThings 0.066

NewPath –– WonderfulPeople 0.065

ChangedPriorities –– DiscStronger 0.064

ChangedPriorities –– BetterHandle 0.058

Intrusions –– Irritability 0.057

BetterSpiritual –– BetterHandle 0.049

AppreciateLife –– NewPath 0.046

BetterSpiritual –– WonderfulPeople 0.043

BetterHandle –– WonderfulPeople 0.041

Closeness –– DiscStronger 0.038

Reactivity –– DiffConc 0.037

AbleDoBetterThings –– StrongerFaith 0.035

BetterSpiritual –– DiffConc 0.031

DiscStronger –– Irritability -0.031

AppreciateLife –– Closeness 0.026

Closeness –– StrongerFaith 0.025

AppreciateLife –– BetterSpiritual 0.024

Intrusions –– Distant 0.024

Intrusions –– DiffConc 0.023

StrongerFaith –– Reactivity 0.022

DiscStronger –– DiffConc -0.022

ChangedPriorities –– Reactivity 0.021

BetterSpiritual –– Avoidance -0.019

WonderfulPeople –– Intrusions 0.018

AppreciateLife –– StrongerFaith 0.017

NewPath –– Reactivity 0.015

WonderfulPeople –– Avoidance 0.014

AppreciateLife –– WonderfulPeople 0.014

AppreciateLife –– Intrusions 0.013

DiscStronger –– Distant -0.013

ChangedPriorities –– Avoidance 0.012

AbleDoBetterThings –– Irritability -0.012

Closeness –– Reactivity 0.011

NewPath –– Irritability -0.009

BetterHandle –– Irritability -0.008

ChangedPriorities –– DiffConc 0

5.5 Edge weight estimates for PTG9+PTSS network (using ggmModSelect algorithm)

Intrusions –– Reactivity 0.458

Irritability –– DiffConc 0.392

AppreciateLife –– AbleDoBetterThings 0.382

Reactivity –– Avoidance 0.299

Distant –– DiffConc 0.298

Closeness –– WonderfulPeople 0.266

NewPath –– BetterHandle 0.254

DiscStronger –– WonderfulPeople 0.23

StrongerFaith –– DiscStronger 0.219

Distant –– Irritability 0.205

Intrusions –– Avoidance 0.201

BetterHandle –– DiscStronger 0.19

BetterSpiritual –– Closeness 0.187

Avoidance –– Distant 0.186

Avoidance –– DiffConc 0.178

Closeness –– NewPath 0.176

StrongerFaith –– WonderfulPeople 0.167

AbleDoBetterThings –– NewPath 0.162

Closeness –– BetterHandle 0.153

BetterSpiritual –– StrongerFaith 0.145

NewPath –– DiscStronger 0.143

AbleDoBetterThings –– BetterSpiritual 0.133

AppreciateLife –– DiscStronger 0.131

AbleDoBetterThings –– DiscStronger 0.128

Reactivity –– Distant 0.126

BetterHandle –– StrongerFaith 0.12

AbleDoBetterThings –– BetterHandle 0.118

Reactivity –– Irritability 0.113

AppreciateLife –– BetterHandle 0.1

AppreciateLife –– NewPath 0.097

AbleDoBetterThings –– Closeness 0.096

Avoidance –– Irritability 0.096

AppreciateLife –– Intrusions 0.09

BetterSpiritual –– DiffConc 0.085

NewPath –– WonderfulPeople 0.08

DiscStronger –– DiffConc -0.078

BetterSpiritual –– Avoidance -0.074

Intrusions –– Irritability 0.072

WonderfulPeople –– Irritability -0.065

NewPath –– Reactivity 0.065

BetterSpiritual –– BetterHandle 0.058

NewPath –– StrongerFaith -0.058

WonderfulPeople –– Avoidance 0.054

WonderfulPeople –– DiffConc 0.053

AbleDoBetterThings –– StrongerFaith 0.051

NewPath –– Irritability -0.045

StrongerFaith –– Reactivity 0.04

6. Raw and scaled centrality and bridge estimates

Raw and scaled centrality and bridge estimates (node strength, expected influence, bridge strength and bridge expected influence) for all estimated networks. NS = Node strength. EI = Expected influence. BS = Bridge strength. BEI = Bridge expected influence.

6.1 Raw and scaled centrality estimates for PTG only network using ggmModSelect algorithm

Raw NS Scaled NS Raw EI Scaled EI

ptg_1 0.74 -0.76 0.61 -1.18

ptg_2 0.91 0.26 0.91 0.35

ptg_3 1.06 1.20 1.06 1.10

ptg_4 0.64 -1.39 0.51 -1.68

ptg_5 0.88 0.08 0.88 0.21

ptg_6 0.96 0.59 0.96 0.61

ptg_7 1.01 0.92 1.01 0.88

ptg_8 0.65 -1.33 0.65 -0.93

ptg_9 1.06 1.20 1.06 1.10

ptg_10 0.75 -0.76 0.75 -0.46

6.2 Raw and scaled centrality and bridge estimates for PTG+PTSS network using ggmModSelect algorithm

Raw NS Scaled NS Raw EI Scaled EI Raw BS Scaled BS Raw BEI Scaled BEI

ptg_1 0.94 -0.18 0.80 -0.56 0.21 1.69 0.21 2.34

ptg_2 0.89 -0.55 0.89 0.16 0.00 -1.15 0.00 -0.32

ptg_3 1.11 1.21 1.05 1.27 0.03 -0.68 -0.03 -0.76

ptg_4 0.83 -1.04 0.55 -2.41 0.16 1.09 0.02 -0.05

ptg_5 0.86 -0.82 0.86 -0.08 0.00 -1.15 0.00 -0.32

ptg_6 1.13 1.31 0.91 0.30 0.10 0.25 0.00 -0.34

ptg_7 1.01 0.38 1.01 1.01 0.00 -1.15 0.00 -0.32

ptg_8 0.78 -1.45 0.67 -1.47 0.04 -0.62 0.04 0.17

ptg_9 1.14 1.39 0.98 0.79 0.08 -0.08 -0.08 -1.32

ptg_10 0.82 -1.14 0.82 -0.38 0.05 -0.50 0.05 0.28

ptss_1 0.89 -0.62 0.89 0.10 0.17 1.13 0.17 1.81

ptss_2 1.08 0.91 1.08 1.51 0.09 0.07 0.09 0.82

ptss_3 1.08 0.90 0.93 0.44 0.12 0.47 -0.02 -0.63

ptss_4 0.82 -1.18 0.82 -0.42 0.00 -1.15 0.00 -0.32

ptss_5 0.96 -0.04 0.79 -0.64 0.09 0.03 -0.09 -1.43

ptss_6 1.08 0.93 0.92 0.37 0.21 1.75 0.06 0.39

6.3 Raw and scaled centrality estimates for PTG network using EBICglasso algorithm

Raw NS Scaled NS Raw EI Scaled EI

ptg_1 0.71 -1.15 0.61 -1.18

ptg_2 0.93 0.27 0.93 0.47

ptg_3 1.06 1.11 1.06 1.16

ptg_4 0.68 -1.31 0.51 -1.74

ptg_5 0.88 -0.03 0.88 0.23

ptg_6 1.01 0.83 0.93 0.51

ptg_7 1.01 0.79 1.01 0.89

ptg_8 0.72 -1.06 0.66 -0.93

ptg_9 1.08 1.29 1.02 0.96

ptg_10 0.77 -0.75 0.77 -0.36

6.4 Raw and scaled centrality estimates for PTG+PTSS network using EBICglasso algorithm

Raw NS Scaled NS Raw EI Scaled EI Raw BS Scaled BS Raw BEI Scaled BEI

ptg_1 0.78 -0.72 0.78 -0.55 0.18 2.38 0.18 2.28

ptg_2 0.90 0.10 0.90 0.31 0.01 -0.73 0.01 -0.16

ptg_3 1.04 1.11 1.02 1.20 0.01 -0.75 -0.01 -0.53

ptg_4 0.56 -2.31 0.52 -2.49 0.05 -0.05 0.01 -0.17

ptg_5 0.87 -0.11 0.87 0.09 0.01 -0.77 0.01 -0.19

ptg_6 0.95 0.48 0.94 0.57 0.02 -0.52 0.01 -0.26

ptg_7 0.99 0.73 0.97 0.86 0.01 -0.83 -0.01 -0.47

ptg_8 0.66 -1.62 0.66 -1.49 0.02 -0.56 0.02 -0.03

ptg_9 1.09 1.46 0.96 0.77 0.07 0.25 -0.07 -1.31

ptg_10 0.78 -0.78 0.78 -0.61 0.03 -0.36 0.03 0.13

ptss_1 0.90 0.08 0.90 0.29 0.18 2.34 0.18 2.24

ptss_2 1.04 1.05 1.04 1.32 0.07 0.34 0.07 0.68

ptss_3 0.98 0.63 0.94 0.60 0.05 -0.13 0.01 -0.23

ptss_4 0.82 -0.45 0.80 -0.45 0.01 -0.74 -0.01 -0.54

ptss_5 0.90 0.12 0.78 -0.55 0.06 0.14 -0.06 -1.23

ptss_6 0.92 0.24 0.88 0.14 0.05 0.02 0.01 -0.21

6.5 Raw and scaled centrality estimates for PTG9+PTSS network (using ggmModSelect algorithm)

Raw NS Scaled NS Raw EI Scaled EI Raw BS Scaled BS Raw BEI Scaled BEI

ptg_2 0.80 -1.05 0.80 -0.43 0.09 0.05 0.09 1.24

ptg_3 1.07 0.86 1.07 1.41 0.00 -1.27 0.00 -0.28

ptg_4 0.68 -1.89 0.53 -2.25 0.16 1.06 0.01 -0.09

ptg_5 0.88 -0.50 0.88 0.10 0.00 -1.27 0.00 -0.28

ptg_6 1.08 0.93 0.87 0.07 0.11 0.35 0.02 0.05

ptg_7 0.99 0.31 0.99 0.88 0.00 -1.27 0.00 -0.28

ptg_8 0.80 -1.05 0.69 -1.22 0.04 -0.68 0.04 0.40

ptg_9 1.12 1.20 0.96 0.68 0.08 -0.12 -0.08 -1.60

ptg_10 0.92 -0.23 0.79 -0.53 0.17 1.26 0.04 0.42

ptss_1 0.82 -0.91 0.82 -0.30 0.09 0.05 0.09 1.24

ptss_2 1.10 1.07 1.10 1.62 0.10 0.27 0.10 1.49

ptss_3 1.09 0.98 0.94 0.52 0.13 0.60 -0.02 -0.62

ptss_4 0.82 -0.95 0.82 -0.33 0.00 -1.27 0.00 -0.28

ptss_5 0.99 0.27 0.77 -0.66 0.11 0.35 -0.11 -2.14

ptss_6 1.09 0.96 0.93 0.44 0.22 1.91 0.06 0.73
